# A Massive Proteogenomic Screen Identifies Thousands of Novel Peptides From the Human “Dark” Proteome

**DOI:** 10.1016/j.mcpro.2024.100719

**Published:** 2024-01-17

**Authors:** Xiaolong Cao, Siqi Sun, Jinchuan Xing

**Affiliations:** 1 Department of Anesthesiology, Zhujiang Hospital, Southern Medical University, Guangzhou, Guangdong, China; 2 Department of Genetics, Rutgers, The State University of New Jersey, Piscataway, New Jersey, USA; 3 Human Genetic Institute of New Jersey, Rutgers, The State University of New Jersey, Piscataway, New Jersey, USA

**Keywords:** proteogenomics, noncanonical open reading frames, novel proteoforms, gene annotation, GTEx

## Abstract

Although the human gene annotation has been continuously improved over the past 2 decades, numerous studies demonstrated the existence of a “dark proteome”, consisting of proteins that were critical for biological processes but not included in widely used gene catalogs. The Genotype-Tissue Expression project generated more than 15,000 RNA-seq datasets from multiple tissues, which modeled 30 million transcripts in the human genome. To provide a resource of high-confidence novel proteins from the dark proteome, we screened 50,000 mass spectrometry runs from over 900 projects to identify proteins translated from the Genotype-Tissue Expression transcript model with proteomic support. We also integrated 3.8 million common genetic variants from the gnomAD database to improve peptide identification. As a result, we identified 170,529 novel peptides with proteomic evidence, of which 6048 passed the strictest standard we defined and were supported by PepQuery. We provided a user-friendly website (https://ncorf.genes.fun/) for researchers to check the evidence of novel peptides from their studies. The findings will improve our understanding of coding genes and facilitate genomic data interpretation in biomedical research.

## Highlights

• Human genome encodes a “dark proteome” of biologically important novel proteins.
• Protein database from 5 million GTEx transcripts and 3.8 million gnomAD variants.
• Identified >170,000 novel peptides with proteomic evidence from >50,000 MS runs.
• User-friendly website to check novel peptide evidence at https://ncorf.genes.fun/.

## Graphical Abstract

**Figure:**
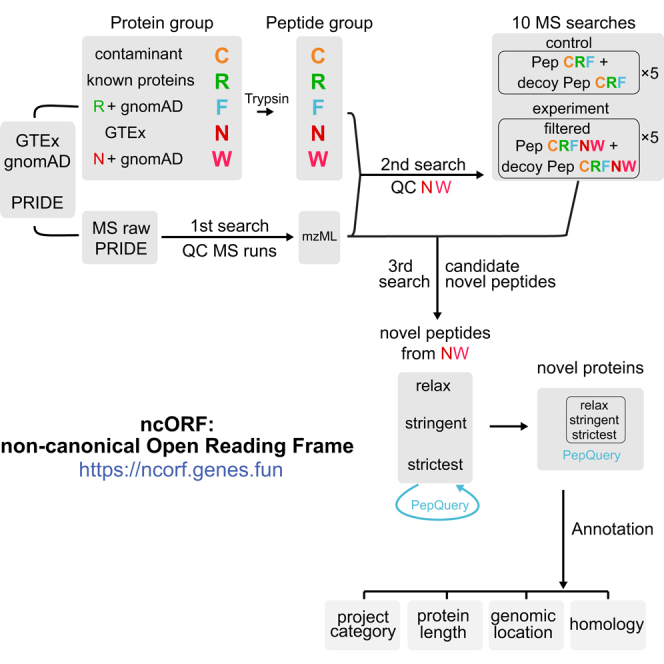

## In Brief

The human genome encodes a “dark proteome” with proteins that are biologically important but not included in widely used gene catalogs. Using a proteogenomic approach, we screened 50,000 mass spectrometry runs to identify novel proteins translated from five million transcripts in the GTEx transcript catalog. We also integrated 3.8 million common genetic variants to improve peptide identification. We identified more than 170,000 novel peptides with proteomic evidence and provided a user-friendly website (https://ncorf.genes.fun/ncorf/) for researchers to check evidence of novel peptides from their studies.

A complete human reference genome and a comprehensive gene annotation are fundamental for biomedical research and clinical diagnosis of genetic disorders. As such, both the reference genome and its annotation require continuous efforts to improve their quality. At the genome level, recently, a complete human reference genome became available with the release of the telomere-to-telomere assembly of the human chromosomes (1, 2). For the gene annotation, GENCODE (3), Ensembl (4), and RefSeq (5) have been actively updating their widely used human gene annotations since the first release of the human reference genome and gene annotation since 2001 (6, 7). In addition, new coding and noncoding transcripts have been constantly identified besides the widely used gene catalogs (8, 9, 10). At the proteome level, the UniProt reference proteome is widely used in proteomic studies (11). However, a high-quality and complete annotation of the human genome is a continuous unfinished task.

Numerous studies suggest the existence of a “dark proteome”, consisting of proteins encoded by noncanonical open reading frames (ncORFs) (reviewed in (12)). For example, nearly 59% of human coding transcripts were found to include upstream ORFs (13), which act as regulators of translation (14). Recently, many of the upstream ORFs have been shown to be protein-coding (15, 16). In addition, a large number of small proteins (*i.e.*, <100 amino acids (AAs)) encoded by unannotated ORFs, which were historically ignored in the annotation of coding sequences (CDSs), were recently confirmed by mass spectrometry (MS)-based proteomics studies (17). These so-called microproteins were shown to regulate multiple processes, including cell proliferation and stem-cell renewal (18, 19, 20). Furthermore, hundreds of proteins translated from long non-coding RNAs (21) and circular RNAs (circRNAs) (22) were identified, and many showed cancer-promoting or inhibiting potential (23). Collectively, these studies demonstrated that the human genome coded a dark proteome and suggested the importance of studying noncanonical proteins. In this study, we define noncanonical/novel proteins as proteins that are currently not included in the standard gene and protein models.

The proteogenomic method, combining data from proteomics, genomics, and transcriptomics, is particularly useful for novel protein identification (24). For instance, 4387 novel peptides were identified through proteogenomic analysis of breast cancer data. Most of these novel peptides were derived from novel proteins that are not included in widely used gene catalogs (25). In another study, with proteomic data from 95 projects, the OpenProt project identified 48,057 novel peptides based on known genic regions in the RefSeq and Ensembl gene catalogs (26). Furthermore, there were many studies of novel proteins in specific diseases or tissues (12). Nevertheless, of the current proteogenomic studies, some focuses on novel ORFs within annotated genes (*e.g.*, OpenProt) (26), some focuses on small proteins (*e.g.*, SmProt, sORF) (27, 28), and many focus on proteomes of a single disease/phenotype (*e.g.*, breast cancer study) (25). Because many ncORFs were not included in the RefSeq and Ensembl transcripts and many proteomic studies did not include sample-specific transcriptome to predict ncORFs for proteogenomic search, a comprehensive proteogenomic survey of novel proteins in diverse range of tissues, sample types, and disease conditions is still lacking.

To this end, in this study, we performed a comprehensive proteogenomic analysis using several largest genomic, transcriptomic, and proteomic datasets to date to identify proteomic-supported novel peptides and proteins, not limiting to annotated genic regions. For tissue-specific transcripts, we used the transcript models from the Genotype-Tissue Expression (GTEx) project. The GTEx project provides high-coverage RNA-seq data from 15,201 samples derived from 49 no-disease tissues of 838 donors of different ages and ancestries (29). For genomic variation among individuals, the Genome Aggregation Database (gnomAD) project is currently the largest open-access reference database (30), which provides information on the common variants in human populations that could improve the performance of proteogenomic analysis (31). In this study, we combined 30 million transcripts modeled from the GTEx project and 3.8 million common genetic variants from the gnomAD project to predict possible novel proteins and their variations. We then screened MS projects in the PRoteomics IDEntification (PRIDE) Database from January 1st, 2012 to September 6th, 2020 and selected more than 50,000 proteomic MS runs from 923 PRIDE projects to identify novel peptides derived from the GTEx-predicted proteins with proteomic support. Using these large-scale data, we identified thousands of novel peptides with different quality control (QC) standards. We provided an interactive webpage (https://ncorf.genes.fun/), serving as an additional resource for researchers to check the existence of novel proteins and study the function of protein of their interest.

## Experimental Procedures

### Known Human Gene Catalogs and Protein Sequences

Common contaminant protein sequences were acquired from the MaxQuant (v1.6.3.4) (32), referred to as protein group C in the text. Known human protein sequences were downloaded from GENCODE release 36 (3), RefSeq release 109.20200815 (5), Ensembl release 102 (4), CHESS 2.2 (8), Human leukocyte antigen sequences, and UniProt human reference proteome sequences (33) (see supplemental Table S1 for download links). The genome sequences, GTF file of gene annotation, protein sequences, and other required files were downloaded accordingly. Protein sequences from the six resources are referred to as protein group R (known human proteins).

### Generation of the GTEx Gene Catalog

The gene model prototype (referred to as the GTEx catalog in the text) in CHESS (8) was used for identifying ncORFs and novel proteins in this study. This GTEx gene catalog was originally generated with about 900 billion RNA-seq sequencing reads from 9795 RNA-seq samples in the GTEx project. The final CHESS catalog was generated from this catalog after a series of filtering and was much smaller (29). To eliminate potential transcriptional noise, transcripts with the max Transcripts Per Million values ≥2 among all GTEx RNA-seq samples and detected in ≥3 samples were kept (referred as the filtered GTEx catalog in the text). TransDecoder (version 5.5.0) (https://github.com/TransDecoder/TransDecoder) was used to identify coding sequences from transcripts of the filtered GTEx gene catalog. To include microproteins translated from ncORFs, the minimum protein length was set to 30. CD-HIT (v4.8.1) (34) was used to recognize proteins which were identical or part of longer proteins. Python scripts were then used to extract the nonredundant protein sequences. Proteins generated in this catalog are referred to as protein group N (GTEx proteins).

### Generation of Protein Groups Considering Population-Specific Alleles

To incorporate genetic variation among human populations, major alleles of all individuals and in different populations were extracted from the gnomAD database (v3.1) (30), as described previously (31). For each gnomAD population, a common allele is defined as an allele that has the highest AF among all possible alleles for a given variant. PrecisionProDB was used to add common alleles to known protein sequences (protein group R) and GTEx proteins (protein group N) to obtain protein sequences which were referred to as protein group F and W, respectively.

### Generation of the Peptide Groups for Each Protein Group for MS Search

It was unrealistic to perform the MS database search on all potential protein sequences from the known genes, the GTEx gene catalog, and their population variations directly. To limit the memory and computational requirements, *in silico* digestion of the protein sequences with trypsin was performed prior MS database search to reduce the database size by removing the duplicated peptide sequences.

As described above, the proteins were divided into five groups: C, common contaminant proteins; R, known human proteins (GENCODE, RefSeq, HLA, CHESS, UniProt, Ensembl); F, protein group R with gnomAD common alleles; N: GTEx proteins; W: GTEx proteins with gnomAD common alleles. All isoleucine (I) residues in the protein sequences were replaced with leucine (L) as they are isomeric amino acids which generally could not be distinguished by MS.

*In silico* trypsin digestion was performed with in-house Python scripts with trypsin as the enzyme and a maximum of two missed cleavages allowed. Peptide groups C, R, F, N, and W were defined as unique peptides digested from the corresponding protein groups. The peptide length was set between 6 and 40 (≥6 and ≤40) for peptide groups C, R, and F and between 9 and 40 (≥9 and ≤40) for peptide groups N and W based on the Human Proteome Project data interpretation guidelines (version 3.0) (35). Additionally, if a peptide existed in multiple peptide groups, it would be assigned to the first peptide group in the order of C, R, F, N, and W. For example, peptides in peptide group N were digested from protein group N and were not present in peptide groups C, R, or F. The target databases for MS search were combinations of peptides from these five groups. To reduce the variability of false positive matches in tandem mass spectrometry (MS/MS) sequence database search, the averaging strategy was adopted (36). That is, for each target database composed of peptides from the five peptide groups described above, five randomized decoy databases of equal size were created using the mimic tool (https://github.com/percolator/mimic). In-house Python scripts were used to remove peptides in the decoy database that are identical or indistinguishable from peptides in the target database (https://github.com/ATPs/human_novo_protein_2022/blob/main/Python_scripts/generation_of_decoy_database.py).

### Mass Spectrometry Raw Data Downloading and Pre-processing

The publicly available proteomics data deposited to the PRIDE Database (37) between January 1st, 2012 and September 6th, 2020 were scanned, and projects with human as the studying organism were selected. To include large-scale proteomic projects with higher resolution and throughput, MS raw data (*i.e.*, files with the extension name of “raw”) of each project were scanned and projects with ≥5 raw files larger than 500 megabytes were selected. MS raw files were downloaded and converted to mzML files with the msconvert tool of ProteoWizard (3.0.20287) (38). Broken MS raw files that could not be converted were removed. The mzML files were further converted to MGF format with the same tool. Furthermore, an MS file was kept if the activation method was “beam-type collision-induced dissociation”, which is also known as “Higher-energy C-trap dissociation” (HCD), in the mzML files.

### First Round of MS/MS Search to Select MS Runs and PRIDE Projects

To select MS runs from trypsin-digested label-free samples and data acquired in data-dependent acquisition mode, the following analyses and filtering were performed. The mzML files were searched against the GENCODE gene catalog with the Comet MS database search tool (2019.01 rev. 5 or 2020.01 rev. 0) (39). The settings were similar to the previous publication (40): 10 ppm (parts per million) for precursor tolerance, 0.02 Da (Dalton) for MS/MS fragment tolerance, and trypsin as the digestion enzyme with a maximum of 2 missed cleavages allowed. The fixed modification was carbamidomethyl (+57.02146) for all cysteines. The variable modifications were oxidation of methionine (+15.9949), N-terminal acetylation (+42.010565), N-terminal carbamidomethyl (+57.02146), deamidation of asparagine and glutamine residues (+0.98402), oxidation of methionine (+15.9949), and N-terminal conversion of glutamine and glutamic acid to pyro-glutamine (−17.026549, −18.010565). The Comet parameter file was stored on GitHub (https://github.com/ATPs/human_novo_protein_2022/blob/main/comet_params/comet.params.decoy.HCD). The protein database file is the combination of proteins from the GENCODE gene catalog and common contaminant proteins (protein group C).

The accuracy of peptide-spectrum matches (PSMs) was evaluated with Percolator (v3.04) (41). For each MS run, the numbers of spectrums, PSMs (regardless of the q-value), and PSMs with q-value ≤0.01 (*i.e.*, 1% false discovery rate (FDR)) were counted. The percentage of spectrums with PSMs or PSMs with q-value ≤0.01 was calculated. MS runs were kept when the two percentages were ≥60% and ≥10%, respectively, and the number of spectrums was ≥5000. Each PRIDE project was further filtered to require >100,000 PSMs detected, >5 and >20% of MS runs passing the QC in the last step. Projects were also manually inspected to exclude a small number of projects which did not meet the need of our study (*e.g.*, project species was labeled as *Homo sapiens* but samples were from the feces).

### The Second Round of MS/MS Search to Select Candidate Novel Peptides

For MS runs passed the first round of quality filtering, a second round of MS/MS search was carried out to select novel peptide candidates. The target database was the combination of all peptides from the five peptide groups (C, R, F, N, and W), and five distinct decoy databases were generated based on the target databases. The target database was then joined with different decoy databases to generate five distinct search databases which were indexed by Comet prior to the searching against the spectrums to save computational resources. The modifications and other settings were the same as described previously, but the “No_cut” option instead of “Trypsin” digestion was enabled. Each MS run file was searched against the five indexed databases. The accuracy of PSMs was evaluated with Percolator for each MS run search (see GitHub for details).

### The Third Round of MS/MS Search to Identify Novel Peptides

To reduce the search space and improve the performance, another round of search was performed with reduced database for each MS run based on the output of the second round of search. For each run, both target and decoy peptides with PSMs after Percolator QC were preserved. Similar to method described previously (42, 43), each of the paired target or decoy peptides of the preserved peptides were also included to create a database with reduced size. Additionally, five control databases were created with all known peptides (peptide groups C, R, and F) and their decoy peptides. Peptides were joined to create pseudo-protein sequences which were indexed with Comet. The 10 indexed databases were used for database search. To reduce the fluctuation of q-values caused by the small sample size of each MS run, the accuracy of PSMs was evaluated with Percolator on each PRIDE project. Results from each project were then used for identifying novel peptides (next section).

### FDR Control and Identification of Novel Peptides

Three filtering standards (relaxed, stringent, and strictest) were used to select candidate novel peptides (peptide groups N and W) from the Percolator results in the third round of MS/MS search. Each spectrum was searched against 10 indexed databases and its PSMs and peptides were evaluated with Percolator 10 times. In the relaxed standard, a spectrum was considered to match a novel peptide when: (1) it matched with a novel peptide ≥3 times with PSM q-value <0.01 and the peptide q-value <0.01 in databases pepCRFNW + decoy pepCRFNW (1–5); (2) it did not match any known peptides (peptide groups C, R, and F) with PSM q-value <0.01 in the other five databases. In the stringent standard, a spectrum was considered to match a novel peptide when: (1) it matched a novel peptide ≥4 times with PSM q-value <0.001 and the peptide q-value <0.001 in searches against databases pepCRFNW + decoy pepCRFNW (1–5); (2) it did not match any known peptides with PSM q-value <0.01 in searches against the other five databases. MS runs and PRIDE projects were summarized for each possible novel peptide (posttranslational modifications ignored) under the relaxed and stringent standards. The strictest standard was defined as stringent standard plus novel peptides were identified in ≥3 MS runs and ≥2 PRIDE projects.

### Novel Peptide Identification with PepQuery

PepQuery (v1.6.2) (44) was used to further reduce possible false positives in novel peptides by excluding the spectrum that could match known proteins with unrestricted posttranslational modifications. In each MS run, the novel peptide candidates were extracted based on the relaxed standard, and the corresponding MS/MS spectrums in the MGF format were extracted. The protein groups C and R were used as the reference protein database. The settings of the modifications for novel peptides in PepQuery were consistent with those described previously.

### Location of CDSs of Novel Peptides in the Genome

To determine the possible genomic location of CDSs of peptides and proteins, the transcripts in the GTEx catalog were also translated directly with TransDecoder. The intermediate files, named “longest_orfs.pep” and “longest_orfs.gff3,” included all ORFs longer than 30 AAs and their locations in transcript sequences, respectively. They were used to determine the possible genomic location of CDSs of peptides and proteins. For candidate novel peptides in peptide group W, their original peptides before I->L AA substitution were identified with the annotations provided by PrecisionProDB (31) and used for locating the peptides in the human reference genome. Peptides resulting from frameshift or stop-gain/loss were excluded from the analysis. To identify all possible locations of CDSs of candidate novel peptides in the human genome, transcripts in the unfiltered GTEx gene catalog were translated and the intermediate files were used to determine the location of peptides in transcripts. The locations of CDSs of peptides in the genome were determined by considering both their location in transcripts and the transcript location in the genome.

### Annotation of Novel Proteins and Peptides

To annotate novel proteins, novel proteins were searched against known protein sequences from NCBI nonredundant (nr), UniProt, and SwissProt databases using DIAMOND (v2.0.11) (45) and BLASTP (v2.9.0) (46) (see supplemental Table S1 for download links. See GitHub for details).

Query-subject matches were kept if the identity >35%, e-value <1e^-10^, and bitscore >50. To find homologous proteins for query proteins, the match was kept if matched length/query length >0.8 or if (matched length/query length) × (matched length/subject length) >0.6. The results from three databases were combined and a single best homologous protein was selected for each query protein based on the highest bitscore. The species names were extracted from the protein fasta file and species lineage was added based on the NCBI taxonomy database.

Intrinsically disordered regions in protein sequences were predicted with IUPred3 (47). A disorder score was predicted for each AA residue in the protein sequence. Residues with a score >0.5 were considered disordered. Protein domains were predicted with InterProScan (v5.38) (48) with SignalP (v4.1) (49) and TMHMM (v2.0) (50) to predict signal peptide and transmembrane domain, respectively. circRNA sequences were downloaded from circAtlas 2.0 (51) website, and a 6-frame translation of circRNAs was performed with in-house Python scripts with methionine as the start and a minimum protein length of 30.

## Results

### Overview of the Experimental Design

**Fig. 1:**
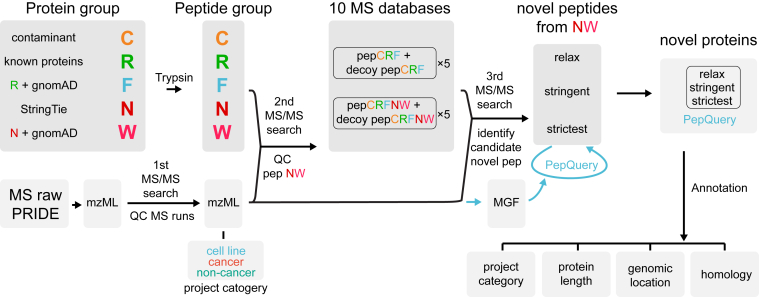
**Overview of the proteogenomic analysis pipeline.** Proteins from different resources were grouped as C, R, F, N, and W (see Fig. 2 for details). Nonredundant peptide groups were generated by *in silico* trypsin digestion. MS raw data were downloaded from PRIDE and converted to mzML format. Three rounds of MS/MS search were performed for the quality control (QC) of mzML files (first search), the QC of peptide groups N and W (second search), and identifying novel peptides (third search). Five MS search databases were generated based on the second MS search for the novel peptide identification in the third round of MS search. Novel peptides were identified with the relaxed, stringent, and strictest standards. PepQuery was run for candidate novel peptides with the matched spectrums in MGF files. Novel proteins were reported by the peptides with/without PepQuery confirmation. Proteins were further annotated for their sample origin (project category), length, genomic location, homology (through BLAST search), etc. MS/MS, tandem mass spectrometry; PRIDE, PRoteomics IDEntification.

**Fig. 2:**
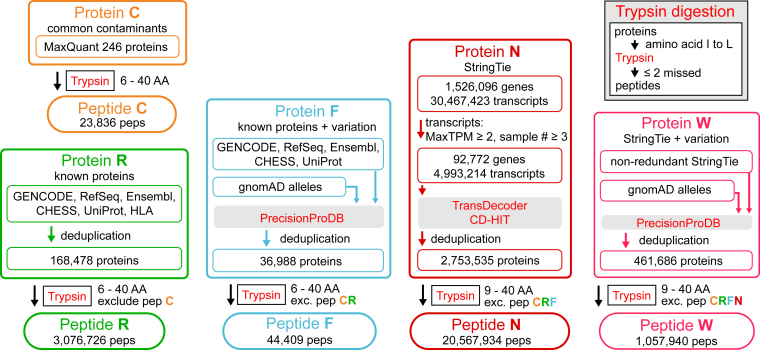
**Preparation of trypsin-digested peptides for MS search.** Proteins from different sources were categorized into five groups: C, R, F, N, and W. Protein group C contains known common contaminants, and group R were unique proteins from known databases. Protein group N was translated from the GTEx StringTie gene model using TransDecoder and CD-HIT. Protein groups F and W were generated by incorporating common alleles from gnomAD projects using PrecisionProDB. Each group of proteins was subjected to *in silico* trypsin digestion procedures (box in the *top right corner*): 1. amino acid isoleucine in proteins was changed to leucine (“I to L”); 2. *in silico* trypsin digestion with a maximum of two missed cleavages. Peptide groups CRFNW were the trypsin digestion products of the corresponding protein groups, with redundant peptides removed. For example, peptide group N included unique peptides digested from protein group N (9–40 amino acids) but not those in peptide groups C, R, or F. MaxTPM: max transcript per million (TPM) values among all samples used in generating the StringTie gene model; sample number: the number of the samples that a transcript is detected from; pep(s): peptide(s); AA(s): amino acid(s); “exc.”: exclude. See Experimental Procedures for details. GTEx, Genotype-Tissue Expression; gnomAD, Genome Aggregation Database.

**Fig. 3:**
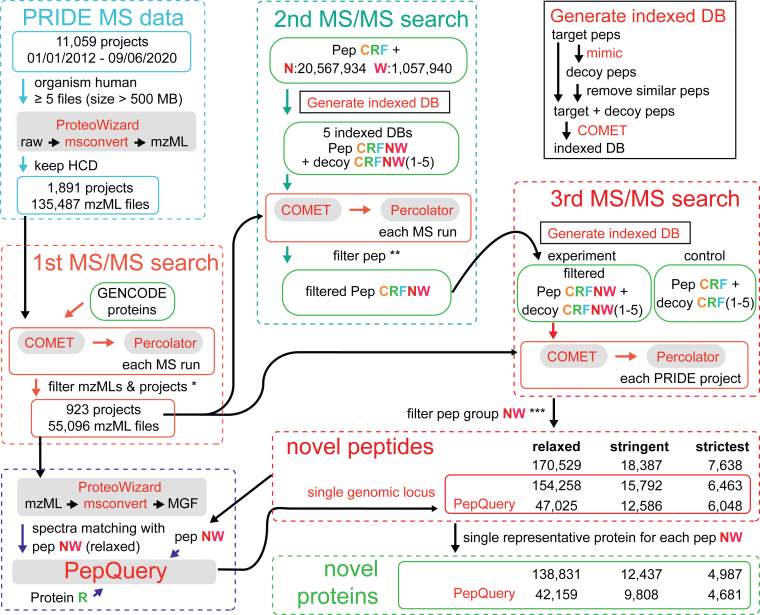
**Proteogenomic analysis pipeline to identify novel proteins.** Projects from PRIDE were filtered to include those with human as the studying organism and contain at least five raw files with a size greater than 500 megabytes (MB). Raw MS data from PRIDE were converted to mzML format, and MS runs with the HCD activation method were kept. mzML files were used for the first MS/MS search against GENCODE proteins with Comet and filtered. Filter mzMLs & projects∗: a mzML file was kept if its spectral counts ≥5,000, its proportion ≥0.6 of spectra with PSMs, and its proportion ≥0.1 of PSMs with FDR <0.01. A PRIDE project was kept if the total counts of PSMs ≥100,000, ≥5 mzML files, and >20% mzML files passed the QC. For the second MS/MS search, five indexed target-decoy peptide databases were created with Comet. The results were used to filter peptides in peptide groups N and W. Filter pep∗∗: for each database search in the second round, both target and decoy peptides with PSM matches were kept. The third MS/MS search was performed with the 10 indexed target-decoy databases and the results were used to identify peptides under the relaxed, stringent, and strictest standards. Filter pep group NW∗∗∗: spectra used to identify novel peptides must match with peptides from peptide groups NW in searching against databases CRFNW + decoy CRFNW and do not match with peptides from control peptide groups CRF in searching against databases Pep CRF + decoy CRF with PSM q-value <0.01. In the relaxed standard, the spectra must match with the same novel peptides ≥3 times with peptide and PSM. Genomic locations of novel peptides were determined based on the 6-frame translation of GTEx StringTie gene models and peptides with a single genomic location were used to determine the novel proteins. PepQuery was run for novel peptides under the relaxed standard to exclude the spectra which could match with unrestricted modifications of known peptides from protein groups CRF. pep(s): peptide(s); HCD: Higher-energy C-trap dissociation. See Experimental Procedures for details. GTEx, Genotype-Tissue Expression; FDR, false discovery rate; MS/MS, tandem mass spectrometry; PRIDE, PRoteomics IDEntification; PSM, peptide spectrum match; QC, quality control.

We adopted a peptide-based proteogenomic analysis pipeline aimed to identify possible novel proteins coded by ncORFs with better FDR control (overview in Fig. 1 and details in Figs. 2 and 3) (52). The input data include (i) known human protein sequences collected from various human gene catalogs, (ii) transcripts predicted from the GTEx project, (iii) common genomic variants extracted from the gnomAD project, and (iv) MS raw datasets from the PRIDE database. Proteins were *in silico* digested with trypsin to obtain peptides that match spectrums from the MS projects. Three rounds of MS search were executed to perform QC for MS data (round 1), QC for peptides (round 2), and to identify possible novel peptides (round 3). We used PepQuery (44), a peptide-centric search engine, for further novel peptide validation. We then applied three different QC standards to reduce possible false positives of identified novel peptides at different confidence levels. All novel proteins were annotated for their sample of origin. We provide a thorough summary of the novel proteins describing their length, genomic location, and homology with known proteins in different species. Because sample-specific RNA-seq data were usually missing for many of the MS projects, the large size of the GTEx RNA-seq data was used to model a large number of proteins coded by ncORFs, serving as a universal database to identify novel peptides. However, the large protein databases required too much computational resources and would decrease the sensitivity in the FDR control (52). Therefore, we adopted the peptide-based search and used the averaging strategy to limit computational resource and to increase the statistic power of FDR control (36). In what follows, we describe in detail the novel proteins and the methodology used to identify them.

### GTEx and gnomAD Data Greatly Increased the Pool of Novel Peptide Candidates for the MS Search

A database, typically consisting of all possible proteins which may be detected in the MS run, is required for novel protein identification through the proteogenomic approach. GENCODE, RefSeq, Ensembl, and CHESS projects all aim to annotate features in the human genome with each currently describing about 60,000 genes, 200,000 transcripts, and 100,000 proteins (supplemental Table S1). In contrast, the GTEx gene catalog, which was assembled from about 900 billion RNA-seq reads collected by the GTEx project, predicts the existence of 1,526,096 genes and 30,467,423 transcripts (Fig. 2) (8). This catalog contains 25 and 131 times higher numbers of genes and transcripts than the GENCODE catalog, respectively. A large proportion of these transcripts have very low-expression levels, and the chance for them to be coding or their encoded proteins being detected through MS analysis is low. Thus, we filtered the transcripts and retained 4,993,214 relatively high expression transcripts to reduce the MS database searching space (Fig. 2, Protein N). We then performed the *in silico* translation of the filtered transcripts. Classic *in silico* translation usually requires a minimum protein length of 100 and one protein per transcript. However, some predicted ncORFs are likely to code for small proteins shorter than 100 AAs and their transcripts may be polycistronic. On the other hand, 3- or 6-frame polycistronic translation is widely used in proteogenomic studies, although the much larger searching space with massive nonreal sequences reduces the sensitivity and specificity (40, 52). To include as many ncORFs as possible while limiting the database size, we performed the translation with TransDecoder, which allowed nonoverlapped polycistronic translation but only include the most likely ORFs, and set the minimum protein length to 30. This procedure gave us 2,753,535 unique proteins (protein group N, Fig. 2), which is about 26 times more than proteins currently annotated in the GENCODE project. The genomic regions spanning the CDS accounted for 1.16% and 11.22% of the human genome in the GENCODE and the filtered GTEx catalogs (supplemental Fig. S1), respectively. Thus, the GTEx catalog provided a rich resource for detecting novel proteins.

We previously showed that incorporating common alleles improves the proteogenomic performance as many alleles in the reference genome were rare in human populations (31). To this end, we used the most common alleles from the gnomAD project (30) together with our PrecisionProDB program to generate alternative protein sequences (Protein groups F and W) based on the known proteins (Protein group R) and GTEx catalog (Protein group N) as input, respectively (Fig. 2).

We used peptides as the search database instead of proteins to save computing resources and to balance the searching space of target and decoy sequences for better FDR control (52). The four groups of proteins along with common contaminants (group C) were digested with trypsin to create five nonredundant peptide groups (Fig. 2). Peptide groups N and W were referred to as novel peptides as they are not present in known peptide groups C, R, and F. Proteins containing novel peptides were referred as novel proteins. Group N contained 20,567,934 peptides, while the gnomAD alleles introduced 1,057,940 additional peptides in group W, making the number of possible novel peptides (peptide groups N and W) about 7 times of known peptides (groups C, R, and F). Collectively, these five groups provide a large database to detect novel peptides.

### PRIDE Constituted a Rich Resource for Novel Protein Identification

To pinpoint which novel peptides from our *in silico* analysis are supported by proteomic evidence, we screened MS projects from the PRIDE database based on the organism, MS activation method, and the number of MS raw files (See Experimental Procedures for details). Specially, we only included high-resolution MS data to limit the candidate search space in MS database search for better accuracy (52). After filtering, we downloaded the raw files from 1891 projects, which included 135,487 MS runs and 5.38 billion spectra. Next, we performed the first round of MS/MS search on all MS runs using the standard GENCODE gene catalog and selected 923 high-quality projects, which included 55,096 MS runs and 2.31 billion spectra (Fig. 3, supplemental Tables S2, and S3).

The 923 projects included a wide variety of research topics, such as cancer, profiling and quantification of proteins, and responses to different stimuli (supplemental Fig. S2*A* and supplemental Table S2). The sample types of these studies included cell lines, cancer tissues or body parts of cancer patients (*e.g.*, breast, lung, ovary, liver, prostate, colon), organs (*e.g.*, brain, heart, lung, liver), and body fluids (*e.g.*, urea, blood plasma or serum, cerebrospinal fluid) of individuals without cancer (supplemental Fig. S2*B*). Based on the source of proteomics data, the projects can be divided into three major categories: cell lines, cancer tissues, and noncancer tissues (supplemental Table S2 and supplemental Fig. S3). The ratio of project counts and MS spectra counts were similar for the three categories (supplemental Fig. S3).

### Two Rounds of MS/MS Searches Identified 170,529 Novel Peptides

The target-decoy approach is widely used as a simple, effective tool for error rate control in MS search (53). However, different decoy databases can lead to discordant results (36) and a large search space can reduce the peptide identification sensitivity (52). Thus, we combined the usage of multiple decoy databases and a two-pass searching strategy and designed a modified peptide-based two-pass searching strategy.

For the 923 projects selected after the first round MS/MS search, we ran the two rounds of MS/MS search with peptides from the peptide groups CRFNW, one with all peptides and one with reduced database. We ran each round the search five times, each time with a distinct decoy database (Fig. 3). To further reduce false positives in peptide identification, we created five control databases with only peptides from known proteins (peptide groups C, R, and F, referred as pepCRF and similar for other abbreviations).

To identify novel peptides, we first excluded PSMs that matched any known peptides (pepCRF) with FDR <0.01 in the five known peptide databases (“Pep CRF + decoy CRF”). From the remaining PSMs, we selected PSMs whose spectra matched novel peptides (peptide groups N and W) and defined three confidence filtering standards: relaxed, stringent, and strictest. For the “relaxed standard”, a spectrum needs to match with the same peptide ≥3 times with FDR <0.01 in the five novel peptide searching databases (“Pep CRFNW + decoy CRFNW”). The relaxed standard was comparable with standards in similar novel peptide identification studies (20, 21, 54, 55). For the “stringent” and “strictest” standards, a spectrum needs to match with the same novel peptide ≥4 times with FDR <0.001 in the five novel peptide searching databases containing novel peptides. The strictest standard also requires that the novel peptides were identified in ≥3 MS runs and ≥2 proteome projects. As a result, we identified 170,529, 18,387, and 7638 novel peptides under the relaxed, stringent, and strictest standards, respectively (Fig. 3 and supplemental Table S4). To further validate novel peptides, we applied PepQuery to identify peptides whose spectra could not match any known peptides with unrestricted modifications (44).

Following the novel peptide identification, we determined the locations of these novel peptides in the genome. More than 90% of the peptides had a single genomic location in the assembled chromosomes and we selected these peptides for further analysis (Fig. 3). The percentages of novel peptides that passed the PepQuery filter were 30.48%, 79.70%, and 93.58% under the relaxed, stringent, and strictest standards, respectively (Fig. 3, n= 47,025, 12,586, and 6,048, respectively). As expected, we observed a strong correlation between the number of detected novel peptides and the number of spectra in each PRIDE project (supplemental Fig. S4*A*). The percentages of relaxed peptides satisfying the strict and strictest standards were similar across all three PRIDE project categories (supplemental Fig. S4*B*).

### Thousands of Novel Proteins Were Detected with Various Standards

The GTEx catalog included a large number of genes and transcripts, many of which with predicted coding capacity. To explain the identified novel peptides with the minimum numbers of genes and their encoded proteins, we selected a single representative protein for each novel peptide by selecting the longest protein with the most matching of novel peptides (See Experimental Procedures for details). As a result, 138,831, 12,437, and 4987 proteins were identified with novel peptide evidence support under the relaxed, stringent, and strictest standards, respectively (Fig. 3 and supplemental Table S5). Many newly discovered proteins were shorter than 100 AAs (referred to as small proteins) based on the previous study (27) and we observed similar results (supplemental Fig. S5*A*). The proportions of small proteins were 73%, 39%, and 30% for relaxed, stringent, and strictest standards, respectively, and reduced to 65%, 33%, and 29%, respectively, if PepQuery filtering was enabled (supplemental Fig. S5*A*).

To determine if novel proteins were generally short because they were from short transcripts, we plotted the length distribution of different transcripts (supplemental Fig. S5*B*). Although the majority of transcripts were less than 500 base pairs in the unfiltered GTEx catalog (supplemental Fig. S5*B*, “all”), most of them were removed in the filtered GTEx catalog. The filtered GTEx catalog showed a comparable length distribution to GENCODE. The transcript lengths of the novel proteins were usually longer than the transcripts in the GENCODE (supplemental Fig. S5*B*). These results suggested that the transcripts of novel proteins were not generally shorter.

### Many Peptides and Proteins Were New Compared with Previous Studies

**Fig. 4:**
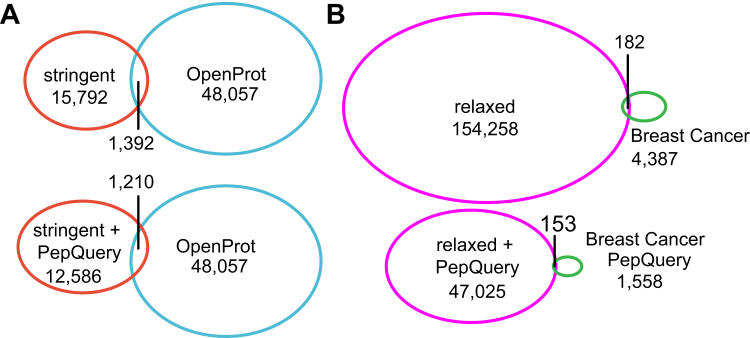
**Comparison with peptides identified in OpenProt and a proteogenomic study of breast cancer.** Peptides with stringent filtering with/without PepQuery filtering were compared with OpenProt (*A*). Peptides with relaxed filtering were compared with the breast cancer study (*B*).

We compared our results with several previous studies (Fig. 4, online resource in Zenodo). The OpenProt Database included 48,057 novel peptides and 43,311 novel proteins with at least one peptide support (release 1.6, alternative proteins and isoforms) (26). We compared our peptides passing the stringent filtering as this standard is the closest to the standards used in OpenProt study and found that less than 10% of the stringent peptides were identified in OpenProt (Fig. 4*A*). We compared the novel peptide sequences identified in proteogenomic analysis of breast cancer data (25) with our results with similar filtering standard. Only 182 without PepQuery filtering and 153 peptides with PepQuery filtering were shared between the two studies, respectively (Fig. 4*B*). The SmProt database stores small proteins identified using Ribo-Seq data (27). Of the 5574 proteins with MS evidence from SmProt, only 25 peptides were identified in the relaxed peptide with single genomic location in our dataset. In conclusion, the vast majority of the novel peptides and proteins were unique in this study. The percentage of shared peptides was low among different studies, probably due to the differences in data used for analyses, data processing pipeline, and filtering standards.

To facilitate the utilization of findings from these studies, we developed a website (https://ncorf.genes.fun/) that allows researchers to verify whether their newly identified novel proteins are supported by MS evidence. When a user inputs a protein sequence, the website will return if the protein includes novel peptides with proteomic evidence. We also included novel peptides identified in OpenProt (26) and the Breast Cancer study (25) in the output.

### Annotation Showed Diverse Origins of Novel Peptides and Proteins

**Fig. 5:**
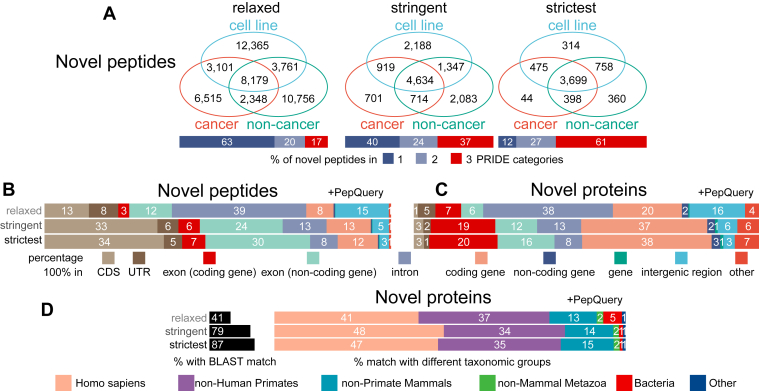
**Annotation of novel peptides and proteins.** Peptides/proteins were identified based on the relaxed, stringent, and strictest standards. Only peptides passed the PepQuery QC were used. *A*, Venn diagrams show the number of peptides identified in different PRIDE sample categories. Cancer: body parts from patients with tumors or cultured cells to study cancer mechanisms. Noncancer: body parts from individuals without cancer. Cell line: cell lines used to study various biological processes other than cancer mechanisms. The percentages of peptides/proteins present in 1, 2, or 3 PRIDE categories were shown in the horizontal bar at the bottom of each Venn diagram. *B* and *C*, relative location of novel peptides (*B*) and proteins (*C*) in different genomic regions. The relative genomic locations of peptides/proteins were determined in the order listed in the figure: CDS, UTR, exon (coding gene), exon (non-coding gene), intron, coding gene, noncoding gene, gene, intergenic region, and other. “exon (coding gene)”: peptide/protein overlaps both “UTR” and “CDS”; “gene”: overlaps both coding and noncoding genes; “other”: overlaps both gene and intergenic region. Only the first genomic location was counted, that is, peptides grouped as in “CDS” would not be counted as “exon (coding gene)”. *D*, percentage of novel proteins with BLAST-matched homologs and the taxonomy distribution of homologs. A single best homologous sequence was selected for each protein and the species were categorized into six groups shown in the figure. CDS, coding sequence; PRIDE, PRoteomics IDEntification; QC, quality control.

The PRIDE MS sample organism parts could be roughly divided into three categories: cell lines, cancer tissues, and noncancer tissues (supplemental Fig. S3). These three categories represented distinct biological conditions. Eighty percent of peptides were identified in one category under the relaxed standard while 61% of peptides were identified in all three PRIDE categories under the strictest standard (supplemental Fig. S6*A*). PepQuery filtering further increased the chance of a peptide being identified in multiple PRIDE categories (Fig. 5*A*).

We analyzed the genomic location of CDSs of novel peptides and proteins relative to the GENCODE gene catalog (Fig. 5, *B* and *C* and supplemental Fig. S6, *B* and *C*). For novel peptides under the relaxed standard, 66% (102,067 out of 154,258) of their CDSs are located completely inside intron or intergenic regions, and the percentages dropped substantially under stringent and strictest standards, to 22% (3540 out of 15,792) and 12% (778 out of 6463), respectively (supplemental Fig. S6*B*). PepQuery filtering further reduced the percentage of peptide CDS inside nonexonic regions (Fig. 5*B*). Similarly, for novel proteins, under the relaxed standard, 63% of their CDSs (87,986 out of 138,831) located entirely inside intron or intergenic regions. The percentage dropped to 24% (3029 out of 12,437) and 13% (647 out of 4987) with the stringent and strictest standards, respectively (supplemental Fig. S6*C*). Previous studies have shown that protein-coding ncORFs were from noncoding RNAs, UTRs of coding RNAs (56), and truncated or extended splicing forms overlapping canonical ORFs (12, 20). We found that up to 17% (14,415 out of 85,249) of UTRs, 20% (9622 out of 47,022) of long noncoding RNAs, 18% (4649 out of 25,673) of noncoding transcripts of coding genes, and 20% (2008 out of 10,169) of pseudogene transcripts annotated in the GENCODE catalog may contain ncORFs coding for proteins (supplemental Table S6). Additionally, 12,156 out of 511,723 circRNAs annotated in the circAtlas 2.0 (51) may have coding capacity, as supported by novel peptides (supplemental Table S6).

Next, we performed homology analysis using BLASTP search against known proteins (NCBI nr, UniProt, and SwissProt databases) (Fig. 5*D* and supplemental Fig. S6*D*). With PepQuery filtering, only 41% of the proteins under the relaxed standard had homologous sequences while this number went up to 87% in the strictest standard (Fig. 5*D*). Since the proteins were translated from the human genome and the CDSs of many proteins partly overlapped with the CDSs of known genes, it was not surprising that most homologous matches were from protein sequences of human and nonhuman primates (Fig. 5*D* and supplemental Fig. S6*D*). We checked the domain structures of novel proteins and found that about 4% of proteins were likely secreted proteins as they were predicted with a signal peptide, about 5% with transmembrane domain, and about 30% with disorder regions (supplemental Table S7, online resource in Zenodo).

To further investigate the characteristics of the novel proteins, we plotted their distribution among multiple features, including protein origin from MS project categories, length, genomic locations, and homologous with known proteins (supplemental Figs. S7–S12) and their density in the genome (supplemental Fig. S13). Under the strictest standard, shorter proteins were more likely to be identified in multiple PRIDE project categories than longer ones (supplemental Fig. S7). Proteins shorter than 100 AAs were less likely to have homologs from known species and more likely to have homologs from bacteria than the longer ones, probably because their sequences were less complex (supplemental Fig. S8). The homology of proteins from different MS categories was similar, suggesting the ubiquitous expression of novel proteins in different samples (supplemental Fig. S9). Under the relaxed, stringent, and strictest standards, more than 80% of proteins located in introns, UTRs, and intergenic regions were shorter than 100 AAs and were overall shorter than those from exonic regions (supplemental Fig. S10). The genomic locations of proteins did not affect their detectability across different MS categories (supplemental Fig. S11). Intronic and intergenic novel proteins were less likely with homologs and more likely matching with bacterial proteins than human exonic ones (supplemental Fig. S12), consistent with the finding that proteins from nonexonic regions were mostly shorter than 100 AAs and short proteins were less likely to have a homolog (supplemental Figs. S8 and S10). This explained the observation that the chance was higher of proteins under the relaxed standard similar to bacteria (Fig. 5*D*), as they were likely shorter and from nonexonic regions. Finally, the novel proteins were detected widely across different chromosomes in the human genome, and the density was similar for the relaxed, stringent, and strictest standards and was also comparable to the annotated genes (supplemental Fig. S13).

To further determine the origins of the novel proteins, we examined several novel proteins manually. Some novel proteins were submitted to the public databases, but current gene models did not include them, probably because the evidence for their existence was weak (supplemental Fig. S14, *A*–*C*). supplemental Fig. S14*D* showed an example of a protein inside the intronic region which was similar but not the same as bacterial proteins. supplemental Fig. S14*E* shows an example of a novel protein located 100% inside CDS regions of the H3.3 histone B gene. This protein is from a new splicing site causing the deletion of a single AA. Note it was not likely explained by genetic variants because we did not find variants that could explain these peptides in gnomAD, NCBI dbVar, or TCGA (The Cancer Genome Atlas) databases. Additionally, we identified a protein (ALL_22318013.p1, supplemental Fig. S14*F*) with six novel peptides passing PepQuery QC. It matched with the 40S ribosomal protein S3a from *Callithrix jacchus* and other primates and was 92% identical to the human 40S ribosomal protein S3a (NP_000997.1). It overlapped with the human “RPS3A pseudogene 21” gene, an annotated pseudogene which was a probably coding gene based on the proteomic evidence. Overall, our findings suggest that some newly discovered proteins are of functional importance, thus experimental validation is needed.

### Transposons Contributed to Novel Peptides with Multiple Genomic Loci

In addition to peptides derived from a single genomic locus, we identified 11,993 peptides that matched multiple genomic regions. Compared to novel peptides with single genomic locus, similar trends were observed for peptides with multiple genomic loci: with more stringent filtering standards, they were more likely to be identified in multiple PRIDE categories, and the percentages of novel proteins shorter than 100 AAs decreased (supplemental Fig. S15*B*). The differences were, the percentage of peptides inside non-exonic regions was 41% under the strictest standard, much higher than 12% for single locus peptides (supplemental Fig. S15*C*), and proteins were more likely to have homologs in bacteria (supplemental Fig. S15*D*). This was likely because repetitive sequences, which cover two-thirds of the human genome, were enriched in introns and intergenic regions and proteins from those regions were similar to proteins coded by transposons and bacteria proteins. Among peptides with >10 genomic loci and >10 MS spectra, we found 10 novel peptides matched to the ORF1 and P40 proteins coded by the LINE1 transposon (supplemental Table S8). Two peptides matched to protein “hepatocellular carcinoma-associated antigen HCA25a” (AAM46782.1), which is translated from an SVA retrotransposon (57).

## Discussion

Proteogenomic methods have assisted the identification of numerous proteins translated from ncORFs, which led to the concept of the “dark proteome” (reviewed in (12)). To systematically assess the scale of the dark proteome, we applied the proteogenomic approach, using the GTEx gene model derived from the tremendous amount of RNA-seq data from the GTEx project (8, 29) and millions of common variants from gnomAD (30) to predict millions of peptides which were not included in canonical gene models. We then validated the existence of novel peptides derived from ncORFs using over 50,000 raw MS files from PRIDE (37). As a result, we identified 170,529 novel peptides with both RNA-seq and proteome evidence. Among them, 154,258 peptides were mapped to a single genomic locus and indicated the existence of 138,831 novel proteins translated from ncORFs. These novel proteins with proteomic support provided a valuable source for future functional studies.

We designed a peptide-based three-pass proteogenomics pipeline to make processing the tremendous amount of data computationally feasible and identified a large number of novel peptides with proteomic support. The FDR control is the major challenge of proteogenomics studies, as reviewed in (52). A large inclusive database is usually required in proteogenomic studies, but an inflated database often decreases sensitivity and specificity (52). Our pipeline is designed with an understanding of these factors. First, the database is large enough to include as many real proteins as possible but not exaggerated. Based on the GTEx gene catalog, 33.29% of the genome could contain ORFs (supplemental Fig. S1). However, most of the transcripts are likely to be nonfunctional transcriptional noise (supplemental Fig. S5*B*), so we excluded transcripts with low expression levels. Also, instead of 6-frame translation which may result in a large database with high percentages of nonreal sequences, we predicted nonoverlapping ORFs with TransDecoder, which preserved ncORFs with coding potential from polycistronic transcripts but did not allow the overlapping of ORFs. We also incorporated millions of the most common variants in the human populations from the gnomAD database. Together, the database inflated about 16 times compared to known proteins (Fig. 2), and the CDS accounts for 11.22% of the genome (supplemental Fig. S5*B*). Secondly, we selected diverse high-quality MS raw data from different biological projects and sample resources (supplemental Figs. S2 and S3). The MS search results were not biased by large projects or certain sample types (*e.g.*, cell lines and cancer samples). Thirdly, we used peptides as the database and performed two rounds of MS search to obtain a more accurate FDR estimation. Using peptides directly not only saved an extensive amount of computational power but also balanced the size of the target and decoy database. Finally, we searched against multiple databases to further improve FDR control performance based on the averaging searching strategy (36). We tested several popular MS searching programs and found that only Comet, designed with high speed and efficiency, could process the huge amount of data. With five different decoy databases, we reduced the variability introduced by the decoy databases and the chance of losing a good target PSM when using a single decoy database. In the third MS run, 5 out of 10 databases were used as controls to make sure that the spectrum matched with novel peptides. For novel peptide/protein selection, our relaxed standard is the common setting for similar studies of novel protein identification (21, 22, 40, 58). We applied two more stringent filtering criteria (stringent and strictest) and used the program PepQuery to further increase the confidence of some novel peptides/proteins. We note that although we tried to increase our confidence of novel peptides, our method identified more novel peptides at the risk of underestimating FDR (42, 59). Researchers will need to use other methods to validate the novel peptides of their interest when necessary.

We found that novel peptides were widely identified in different resources, including cell lines, cancer, or normal tissues, and confirmed that exons of annotated genes (coding or non-coding, Fig. 5*A*) were more likely to contribute to novel peptides than nonexonic regions. With more stringent filtering standards, more novel peptides passed the PepQuery validation, were identified in multiple PRIDE sample categories, and were located inside exonic regions (Fig. 5). The percentage of proteins that are longer than 100 residues and have homologous sequences was also higher (Fig. 5). We proposed the mechanisms below to explain these trends.

The vast majority of the human genome is pervasively transcribed (20, 60, 61), leading to the large number of transcripts in the GTEx gene catalog. Due to transcription noise, most of these transcripts are short, rare, and noncoding (supplemental Fig. S5*B*). Translational noise also exists widely inside the cell (62), producing proteins which are likely short, rare, and short-lived. Thus, most predicted peptides in peptide groups N and W were not detected, and the detected ones under the relaxed standard were mostly identified in a single MS project category, and their proteins were short. Since the majority of these transcripts were from introns and intergenic regions which occupy more than 95% of the genome, many detected novel peptides located in the introns and intergenic regions. Sequences in introns and intergenic regions are typically less conserved, and proteins translated from them are more likely to be short and have a simple structure. This was also confirmed by the findings that proteins from small ORFs are less conserved (12) and proteins coded by ncORFs are more disordered and unstable than canonical proteins (55). Novel peptides from these regions are more likely to be similar to each other and with less complex AA sequences, leading to more similar decoy peptides, relatively larger FDR values, and a lower chance of passing PepQuery QC. Their proteins also have a lower probability of finding a homolog in protein databases. As expected, novel peptides and proteins identified under the relaxed standard were overall more disordered than those under the stringent and strictest standards (supplemental Fig. S16, *A* and *B*). Not surprisingly, for peptides mapped to multiple genomic loci, the peptides and proteins were slightly more disordered than those mapped to a single genomic locus (supplemental Fig. S16, *C* and *D*).

One interesting finding is that some proteins had the best match with bacteria sequences in the NCBI nr database. Although there could be bacteria contamination in the GTEx RNA-seq data, the GTEx gene catalog is based on the human reference genome GRCh38, which does not contain large fragments of bacteria sequence (2). There are likely bacterial contamination in the proteomics datasets, but we have included common contamination sequences in the database, and other bacteria sequences were not included in the target database for the MS search. Taken together, it is unlikely that these sequences were due to bacteria contaminations. Another potential explanation is the protein/peptide differences in the NCBI database in different species. There are many predicted bacteria protein sequences in the NCBI nr database based on the simple ORF rules, and these transcripts were not annotated as coding in mammalian genomes. Because our sequences with bacteria hits were mostly only passed the relaxed filtering, short, and translated from introns or intergenetic regions, they will have a higher chance matching the predicted small peptides from bacteria studies in the NCBI database.

Although we identified a large number of novel proteins, more noncanonical proteins remained undiscovered due to the limited sensitivity of current methods and techniques. While we used the database search method, *de novo* peptide sequencing offers another promising approach to identify novel peptides. Despite its technical challenges, we anticipate further improvements and increased utilization of this method for novel peptide identification. *De novo* peptide sequencing enables the identification of peptides absent in the database, which can come from ncORFs, somatic mutations, or RNA-editing events (63). Furthermore, single-molecule protein sequencing techniques have the potential to the change current large-scale protein detection methods dramatically (64, 65). With these techniques and improved references, we envision the annotation of human coding genes would be continuously improved.

## Data availability

MS raw data were downloaded from the PRIDE database. Data used in this study, including the GTEx gene catalog, are available at Zenodo (https://zenodo.org/records/10417233). Explanations of the files at Zenodo and some codes for data processing and processed data are available on GitHub (https://github.com/ATPs/human_novo_protein_2022). We provided a website for users to check the existence of their proteins coded by ncORFs (https://ncorf.genes.fun).

## Supplemental data

This article contains supplemental data.

## Conflict of interest

The authors declare that they have no conflicts of interest with the contents of this article.
