# Supplementary material for: A Massive Proteogenomic Screen Identifies Thousands of Novel Peptides From the Human “Dark” Proteome
Source: Mol Cell Proteomics. 2024 Jan 17;23(2):100719. doi: 10.1016/j.mcpro.2024.100719 (PMC10867589; doi:10.1016/j.mcpro.2024.100719)
Supplement: Supplemental Figures [file mmc9.pdf]

Fig. S1

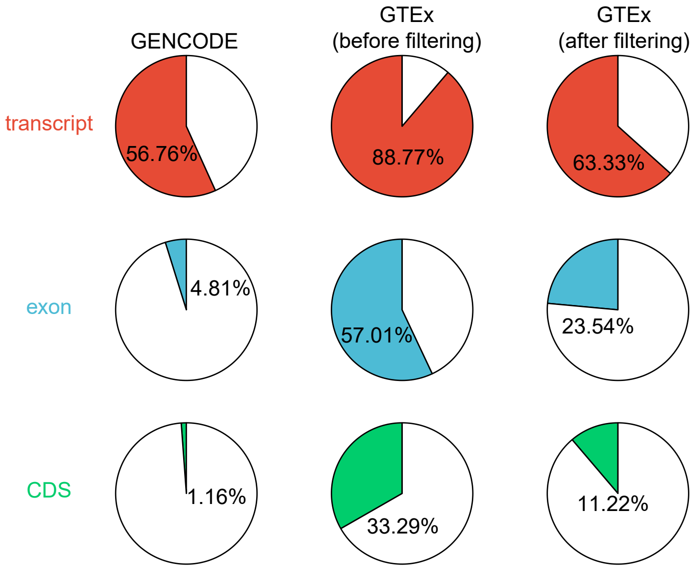
GENCODE
GTEx
(before filtering)
GTEx
(after filtering)
transcript
exon
CDS
56.76%
88.77%
63.33%
4.81%
57.01%
23.54%
1.16%
33.29%
11.22%

Fig. S2

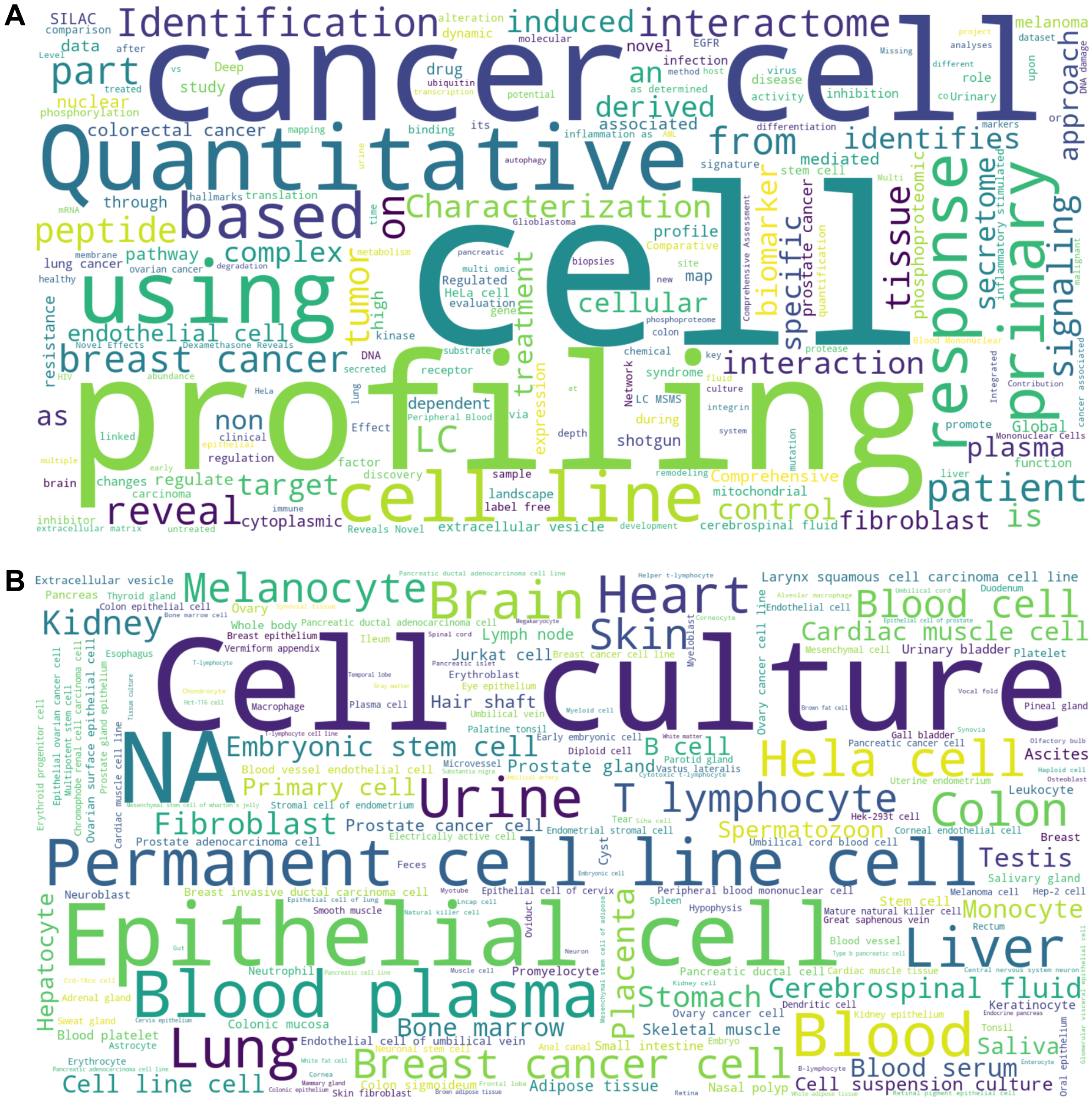
A
B
cancer
cell
Quantitative
profiling
using
based
cell line
response
primary
Identification
interactome
induced
Characterization
peptide
complex
breast cancer
endothelial cell
reveal
target
patient
fibroblast
plasma
signaling
secretome
tissue
biomarker
specific
identifies
from
derived
part
interaction
cellular
treatment
tumor
approach
control
cytoplasmic
extracellular vesicle
Comprehensive
shotgun
dependent
non
as
LC
melanoma
Cell culture
NA
Permanent cell line cell
Epithelial cell
Blood plasma
Lung
Breast cancer cell
Urine
T lymphocyte
Hela cell
Colon
Heart
Brain
Melanocyte
Blood cell
Cardiac muscle cell
Skin
Kidney
Liver
Blood
Placenta
Stomach
Cerebrospinal fluid
Embryonic stem cell
Fibroblast
Primary cell
Hepatocyte
Bone marrow
Blood serum
Saliva
Testis
Monocyte
Spermatozoon
Cell line cell
Cell suspension culture
Skeletal muscle
Prostate cancer cell
Prostate gland
B cell
Hair shaft
Jurkat cell
Lymph node
Adipose tissue
Small intestine
Colonic mucosa
Ascites
Leukocyte
Keratinocyte

Fig. S3

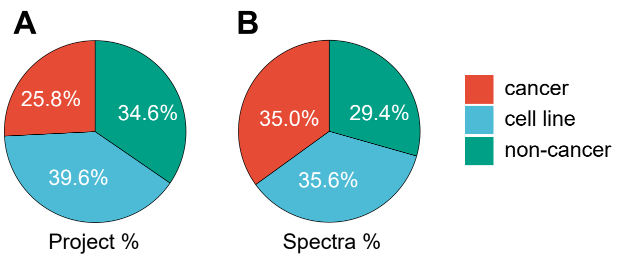

A
25.8%
34.6%
39.6%
Project %
B
35.0%
29.4%
35.6%
Spectra %
cancer
cell line
non-cancer

Fig. S4

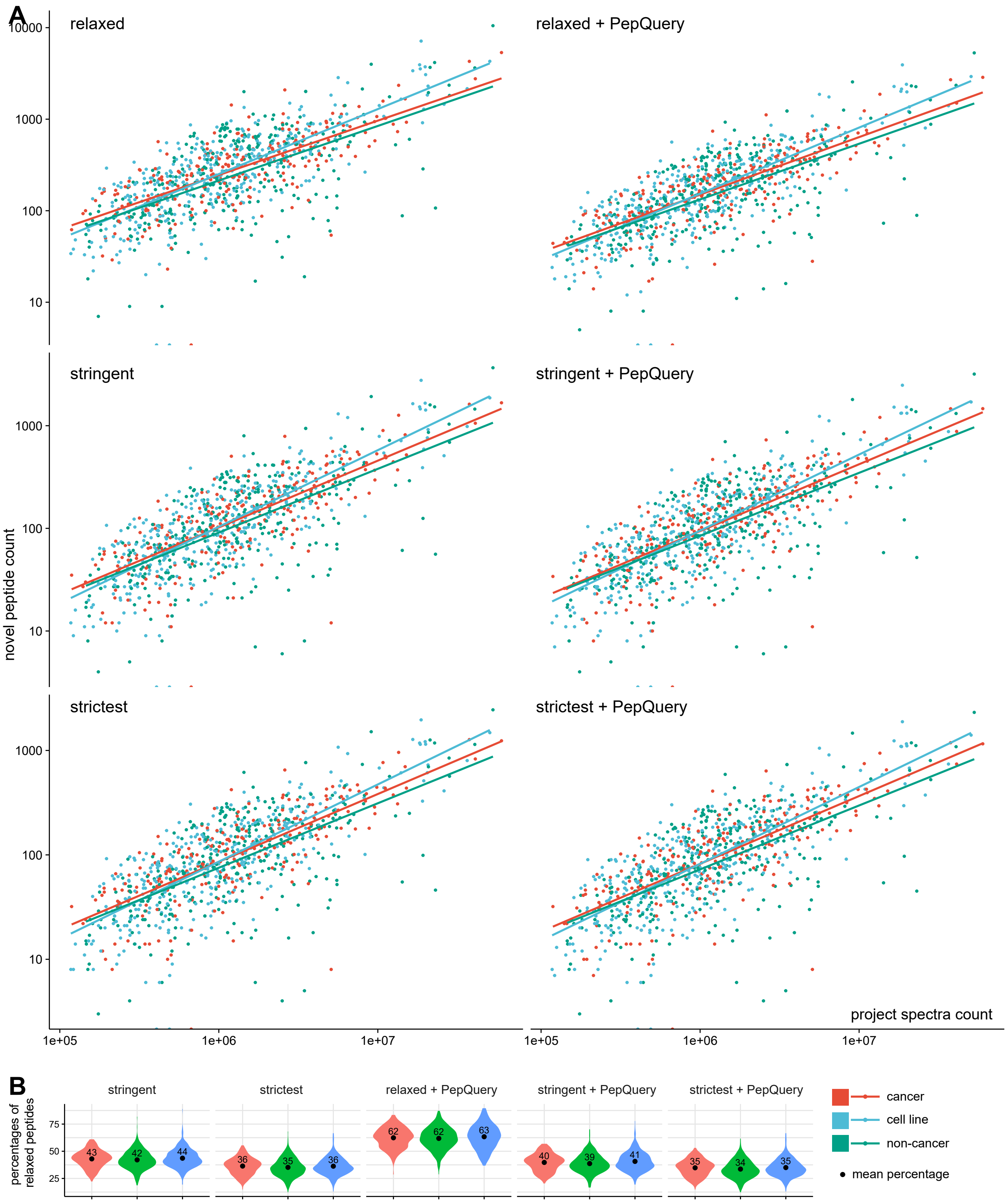
A
relaxed
relaxed + PepQuery
stringent
stringent + PepQuery
strictest
strictest + PepQuery
10000
1000
100
10
novel peptide count
project spectra count
1e+05
1e+06
1e+07
B
percentages of relaxed peptides
stringent
strictest
relaxed + PepQuery
stringent + PepQuery
strictest + PepQuery
75
50
25
43
42
44
36
35
36
62
62
63
40
39
41
35
34
35
cancer
cell line
non-cancer
mean percentage

Fig. S5

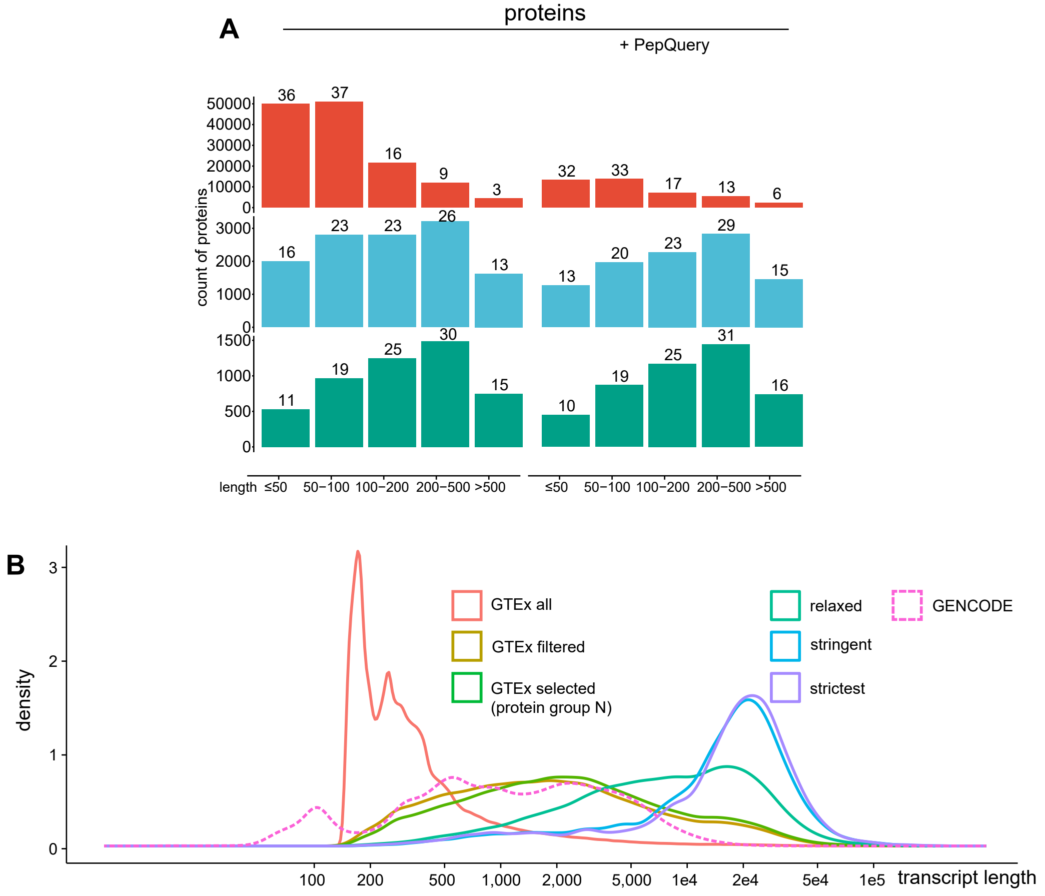
A
proteins
+ PepQuery
count of proteins
36
37
16
9
3
32
33
17
13
6
16
23
23
26
13
13
20
23
29
15
11
19
25
30
15
10
19
25
31
16
length ≤50 50−100 100−200 200−500 >500
≤50 50−100 100−200 200−500 >500
B
density
GTEx all
GTEx filtered
GTEx selected
(protein group N)
relaxed
stringent
strictest
GENCODE
100 200 500 1,000 2,000 5,000 1e4 2e4 5e4 1e5
transcript length

Fig. S6

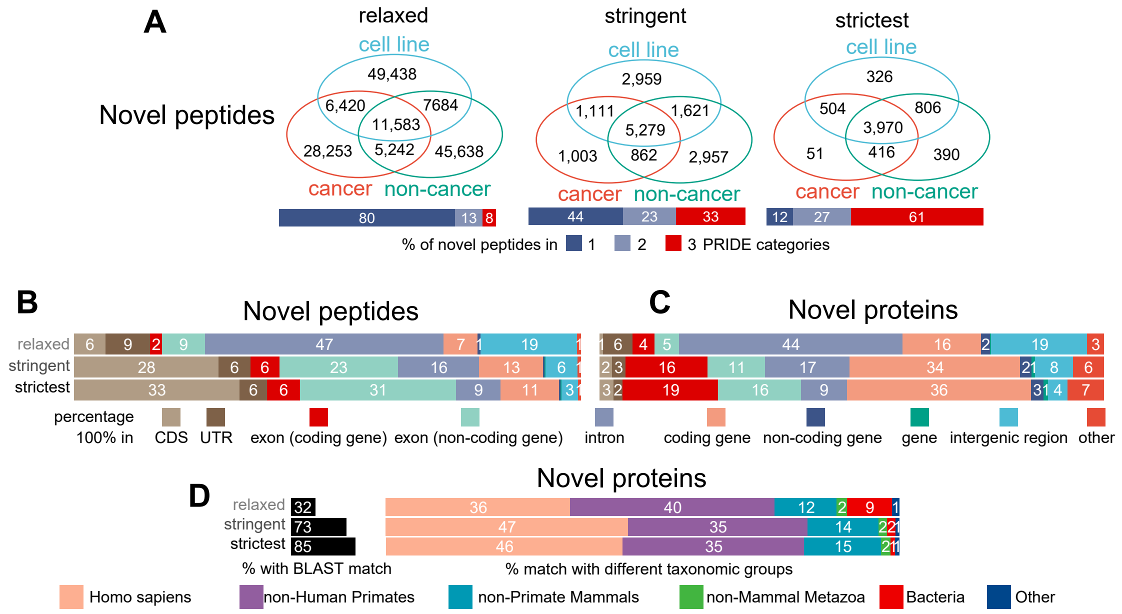
A
Novel peptides
relaxed
cell line
49,438
6,420
7684
11,583
28,253
5,242
45,638
cancer
non-cancer
80
13
8
stringent
cell line
2,959
1,111
1,621
5,279
1,003
862
2,957
cancer
non-cancer
44
23
33
strictest
cell line
326
504
806
3,970
51
416
390
cancer
non-cancer
12
27
61
% of novel peptides in 1 2 3 PRIDE categories
B
Novel peptides
relaxed
stringent
strictest
C
Novel proteins
percentage 100% in
CDS
UTR
exon (coding gene)
exon (non-coding gene)
intron
coding gene
non-coding gene
gene
intergenic region
other
D
Novel proteins
relaxed
stringent
strictest
32
73
85
% with BLAST match
% match with different taxonomic groups
Homo sapiens
non-Human Primates
non-Primate Mammals
non-Mammal Metazoa
Bacteria
Other

Fig. S7

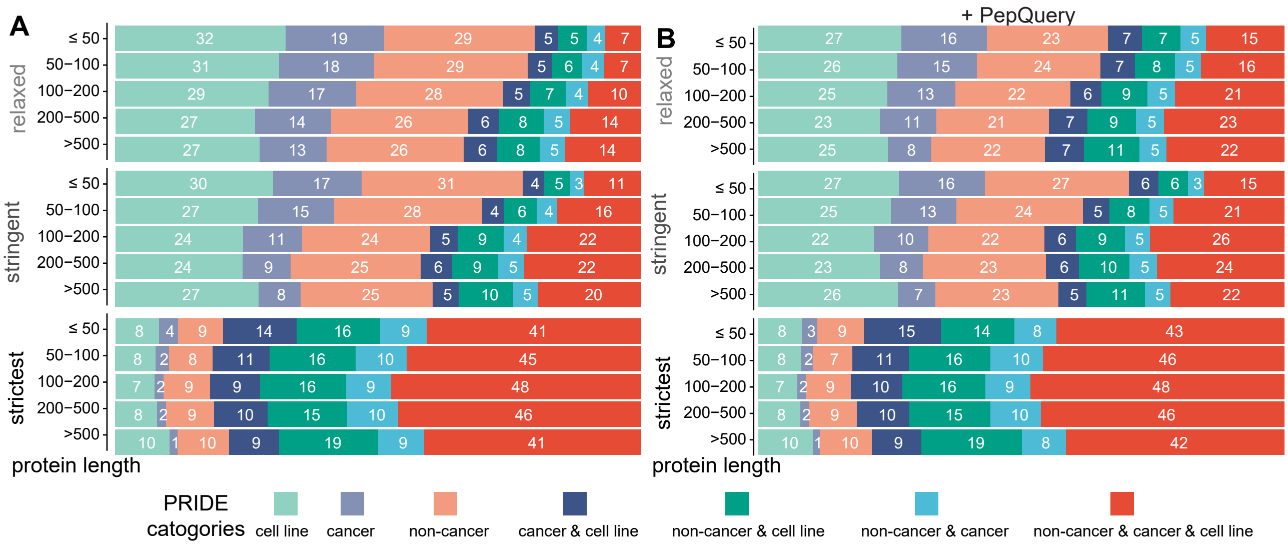

A
B
+ PepQuery
relaxed
stringent
strictest
≤ 50
50−100
100−200
200−500
>500
protein length
PRIDE catogories
cell line
cancer
non-cancer
cancer & cell line
non-cancer & cell line
non-cancer & cancer
non-cancer & cancer & cell line

Fig. S8

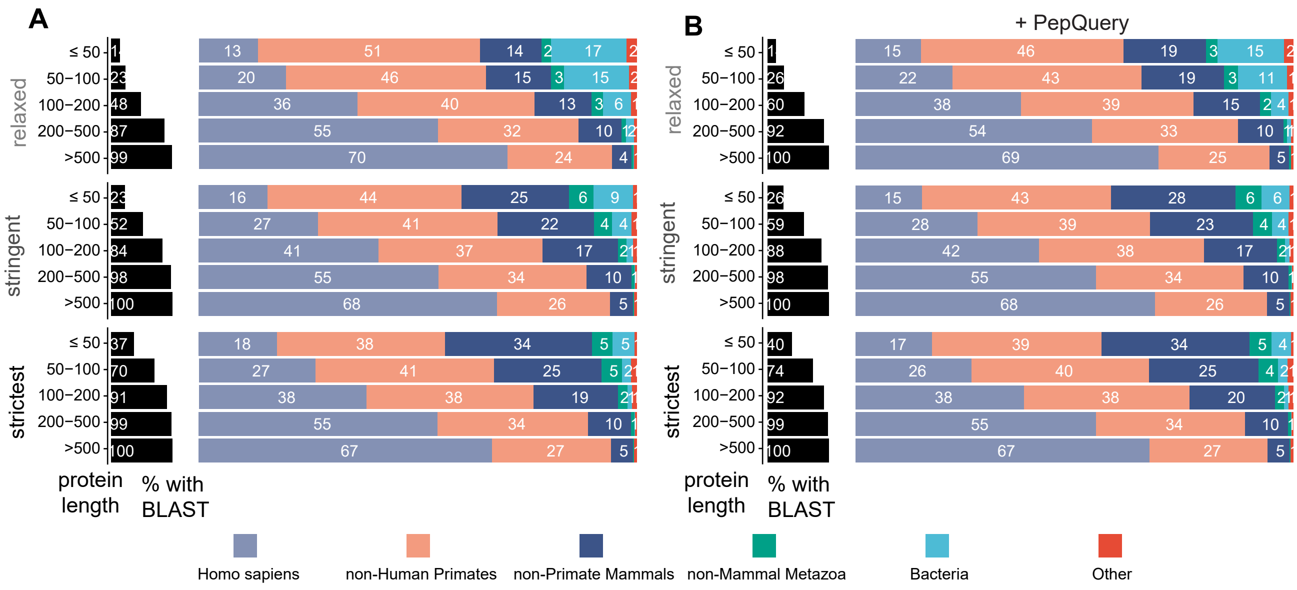
A
B
+ PepQuery
relaxed
stringent
strictest
≤ 50
50−100
100−200
200−500
>500
protein length
% with BLAST
Homo sapiens
non-Human Primates
non-Primate Mammals
non-Mammal Metazoa
Bacteria
Other

Fig. S9

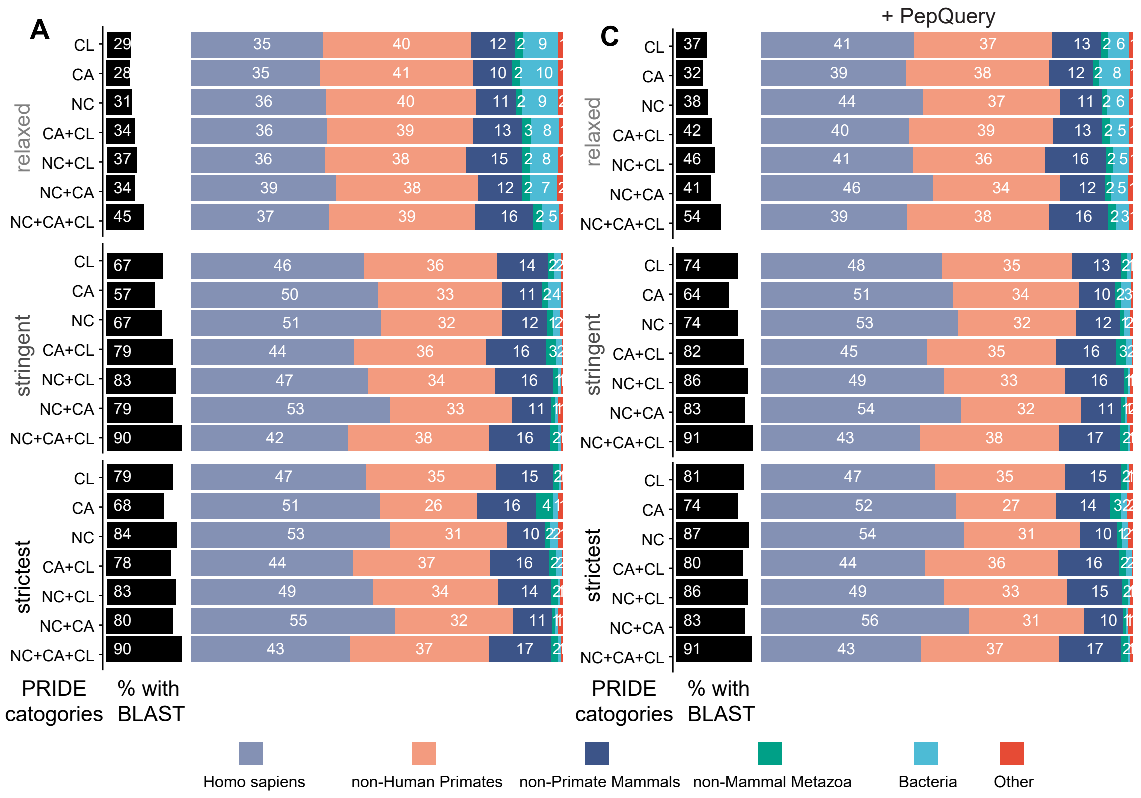
A
C
+ PepQuery
relaxed
stringent
strictest
CL
CA
NC
CA+CL
NC+CL
NC+CA
NC+CA+CL
PRIDE catogories
% with BLAST
Homo sapiens
non-Human Primates
non-Primate Mammals
non-Mammal Metazoa
Bacteria
Other

Fig. S10

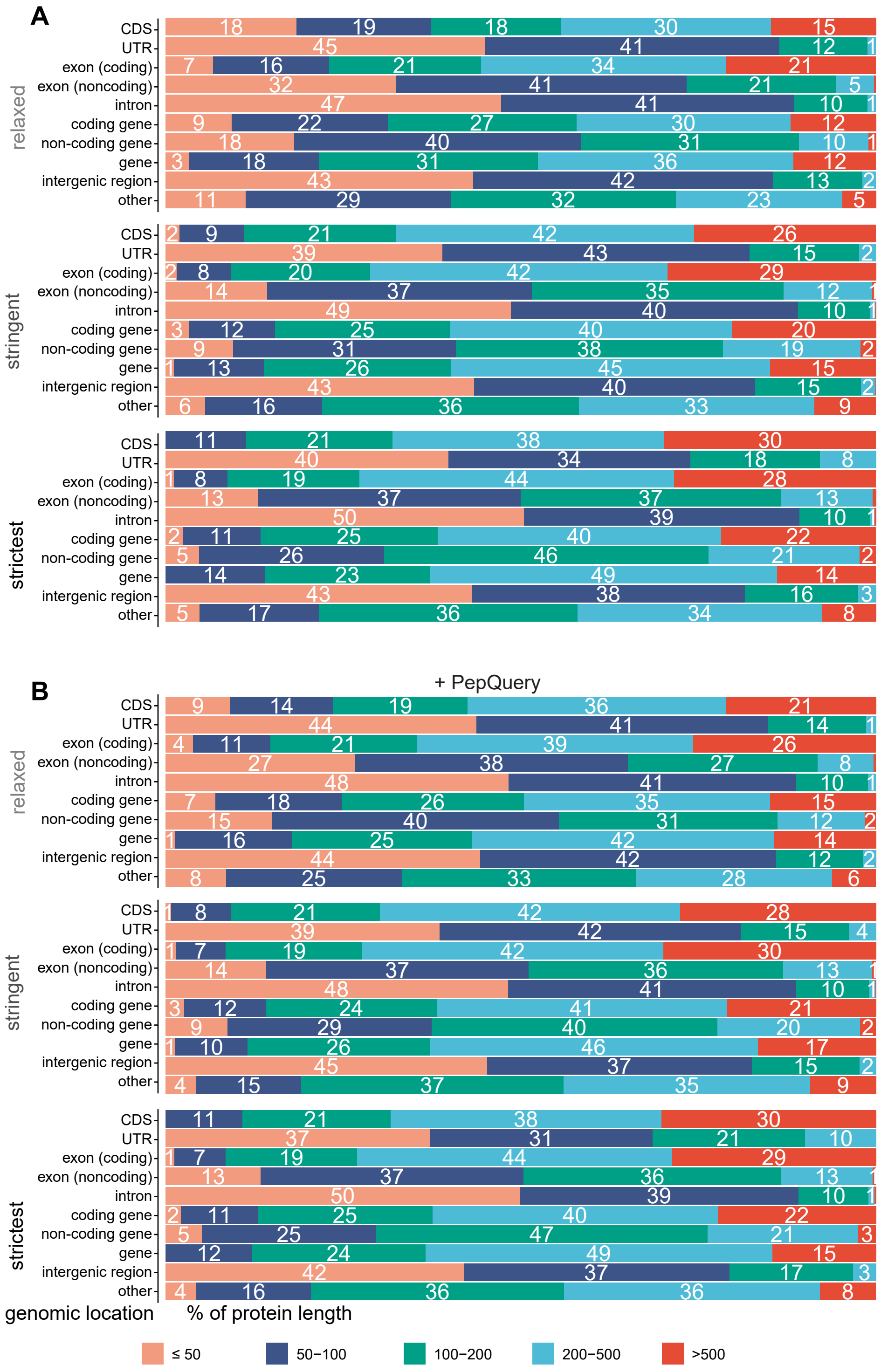
A
B
+ PepQuery
relaxed
stringent
strictest
CDS
UTR
exon (coding)
exon (noncoding)
intron
coding gene
non-coding gene
gene
intergenic region
other
genomic location
% of protein length
≤ 50
50−100
100−200
200−500
>500

Fig. S11

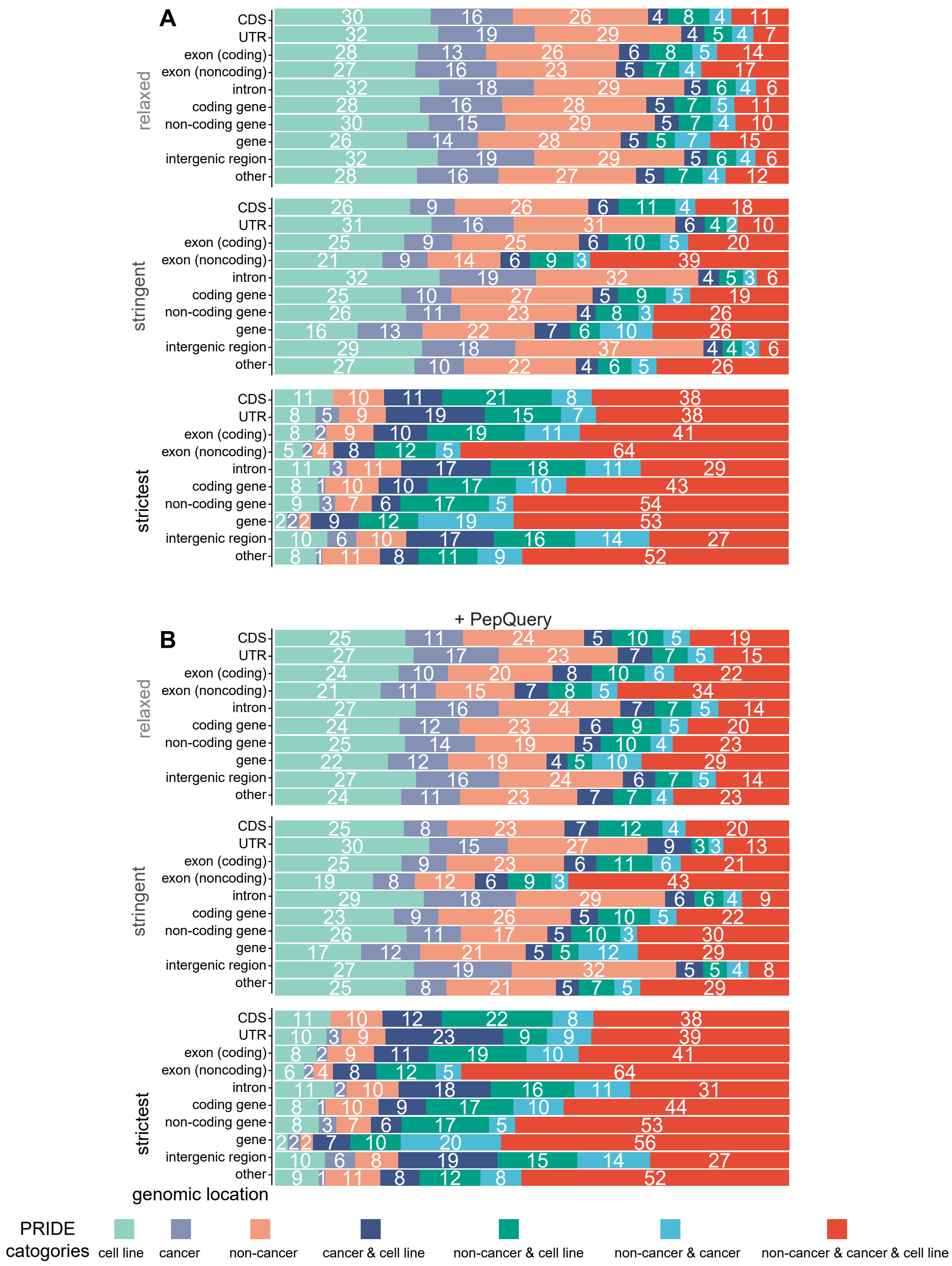
A
B
+ PepQuery
relaxed
stringent
strictest
CDS
UTR
exon (coding)
exon (noncoding)
intron
coding gene
non-coding gene
gene
intergenic region
other
genomic location
PRIDE catogories
cell line
cancer
non-cancer
cancer & cell line
non-cancer & cell line
non-cancer & cancer
non-cancer & cancer & cell line

Fig. S12

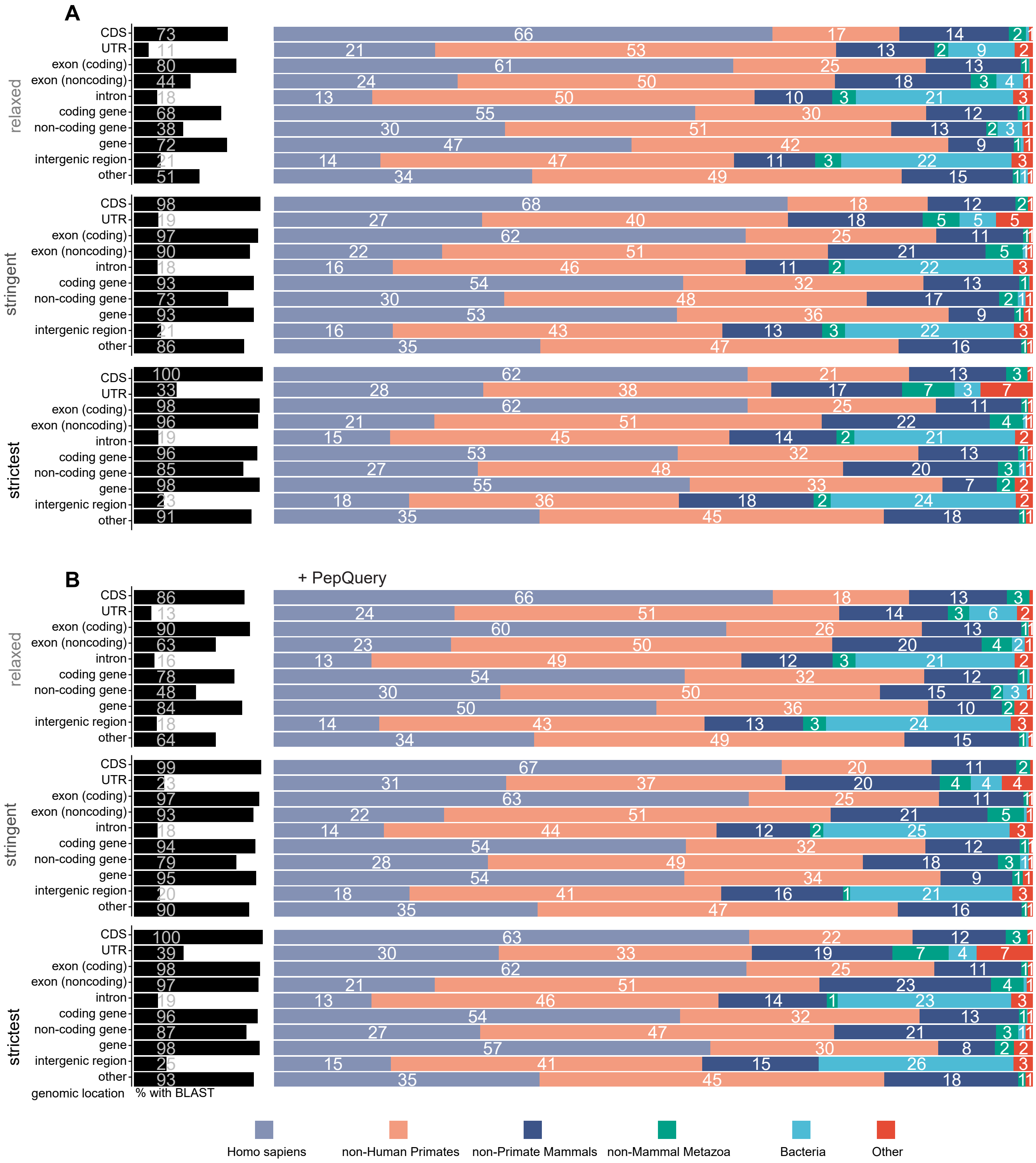
A
B
+ PepQuery
relaxed
stringent
strictest
CDS
UTR
exon (coding)
exon (noncoding)
intron
coding gene
non-coding gene
gene
intergenic region
other
genomic location
% with BLAST
Homo sapiens
non-Human Primates
non-Primate Mammals
non-Mammal Metazoa
Bacteria
Other

Fig. S13

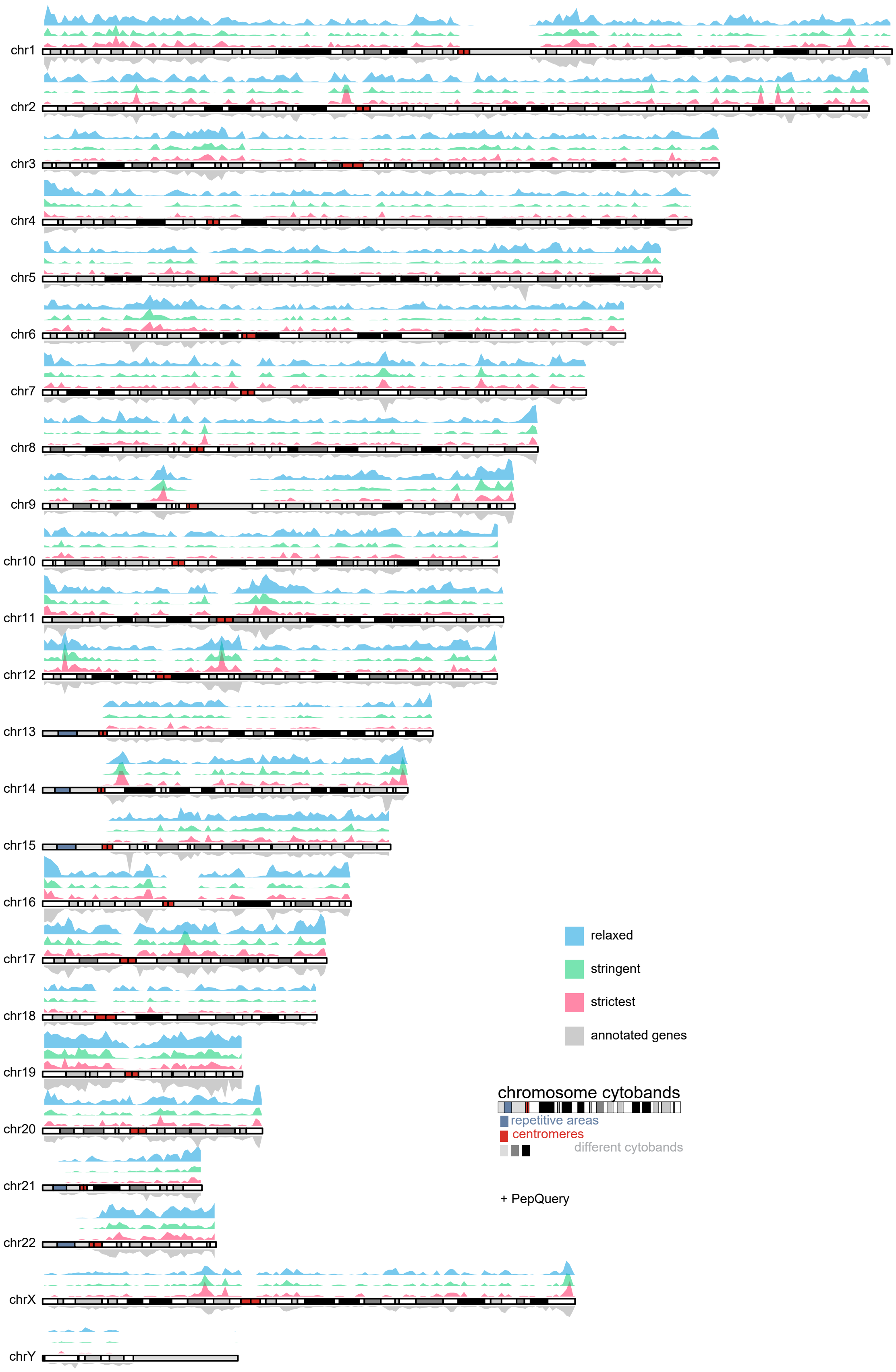
chr1
chr2
chr3
chr4
chr5
chr6
chr7
chr8
chr9
chr10
chr11
chr12
chr13
chr14
chr15
chr16
chr17
chr18
chr19
chr20
chr21
chr22
chrX
chrY
relaxed
stringent
strictest
annotated genes
chromosome cytobands
repetitive areas
centromeres
different cytobands
+ PepQuery

Fig. S14

A ALL_16330199.p2, 100% inside intergenic region, similar to PZI44384.1, an hypothetical protein from bacteria

MQRRNVRLKHSHRIPTGTLPSGPVRKGSLSFRLQNGRSTDCLPPTPGKPAGTQCRPMK**AAAGAELCQALGQSYTGLWESTPHISMHRIWDMK**

B ALL_04175594.p2, 100% inside UTR, identical to Q9H5C3_HUMAN, an unknown protein from human with evidence at transcript level

MVPLKSYYALPSPAFSRNHTGLRSELRSGQSELSR**TGPAFLHCHVPHAHHSSPAAGQGQNWASPGQGNQK**LFLGEIEPPVAPWSSGGWAAAHSACGQNTCSPLSLSAEQTQGIALLSPGN
FSEILDLASLRPTMPHKGCSGRRGCRRQRVPRRELVAPAPLELLK**ITQQLLLTGMLMPR**KQAQERKAECDRAEPGPGAPGEAFLGLQRSATGGRSPELPI

C ALL_12687366.p1, 100% inside non-coding exons, identical to AAD43569.1, “epstein-barr virus-induced zinc finger protein” from human

MCVGKPSVRAQILFCIRESILGRNHIHVISVAKVSVR**IQTLLNIEGSTLER**NPINVMSVGKLLIRAQSLFYIRGFILERNPIPVINVAKPSVGFQILLIINEFTLERSLTHVISAIKCLV
EDEILLNITEFIQVR**NPMNVMNVGK**PLVRAPTLFFIRESTLERNLMHVVIVLKALVAVQILLSIKEYTLERNHMHVISVIKVLVKAQTSLNIREYTLVKSLIIAIVVRKPSVRVLTLFFI
REFTLEKNYYLCTQCSKSFSQISDLIKHQRIHTGEKPYKCSECRKAFSQCSALTLHQRIHTGKKPNPCDECGKSFSRRSDLINHQKIHTGEKPYKCDACGKAFSTCTDLIEHQKTHAEEK
PYQCVQCSRSCSQLSELTIHEEVHCGEDSQNVMNVRKPLVCTPTLFSTRDTVPEKNLMNAVDY

D ALL_14549922.p3, 100% inside introns, similar to WP_205334836, “DUF1725 domain-containing protein” from Klebsiella pneumoniae

```
 1   MFTAALFTIGKTWNQSKCQSMTDCVKKIWYIYIMEYYAAIKKNEIMSFARTWMKLEAIILSHKSPGAIQRRKQQTLGST   78
     MF AALFTI KTWNQ KC +M D +KK+W+IY +EYYAAIKK+E MSFARTWMKLE IILS  S G
 3   MFIAALFTIAKTWNQPKCPTMIDWIKKMWHIYYLEYYAAIKKDEFMSFARTWMKLETIILSKLSQGQ              69
```

E ALL_11965551.p2, 100% inside CDS of ENSG00000132475 gene, identical to XP_010576034.1 “histone H3.3 isoform X2” from Haliaeetus leucocephalus.
Below is match with human protein P84243 coded by ENSG00000132475 “Histone H3.3”

```
                                                                                                     FQSAAIGALQ-ASEAYLVGLFEDTNLCAIHAK
                                                                                                     FQSAAIGALQ-ASEAYLVGLFEDTNLCAIHAKR
                                                                                                 TDLRFQSAAIGALQ-ASEAYLVGLFEDTNLCAIHAK
1    MARTKQTARKSTGGKAPRKQLATKAARKSAPSTGGVKKPHRYRPGTVALREIRRYQKSTELLIRKLPFQRLVREIAQDFKTDLRFQSAAIGALQ-ASEAYLVGLFEDTNLCAIHAKRVTI   119
1    MARTKQTARKSTGGKAPRKQLATKAARKSAPSTGGVKKPHRYRPGTVALREIRRYQKSTELLIRKLPFQRLVREIAQDFKTDLRFQSAAIGALQEASEAYLVGLFEDTNLCAIHAKRVTI   123

120  MPKDIQLARRIRGERA  135
124  MPKDIQLARRIRGERA  136
```

F ALL_22318013.p1, overlap with gene ENSG00000214784 (RPS3A pseudogene 21) and intergenic region.
Most similar to XP_035156676.1 “40S ribosomal protein S3a-like” from Callithrix jacchus
Below is match with human NP_000997.1, “40S ribosomal protein S3a isoform 1”

```
                                                                                                            NCLSNFHGMDLTR
5    MAVGKNKCLTKGGKKGAKKKVVDQFSKKDWYDVKASAMFSIRNIGKTLVTRTQGTKIASDGLKGRVFEVSLADLQNDEVAFRKFKLITEDVQGKNCLSNFHGMDLTRDKMCSMVKKWQTM  124
     MAVGKNK LTKGGKKGAKKKVVD FSKKDWYDVKA AMF+IRNIGKTLVTRTQGTKIASDGLKGRVFEVSLADLQNDEVAFRKFKLITEDVQGKNCL+NFHGMDLTRDKMCSMVKKWQTM
1    MAVGKNKRLTKGGKKGAKKKVVDPFSKKDWYDVKAPAMFNIRNIGKTLVTRTQGTKIASDGLKGRVFEVSLADLQNDEVAFRKFKLITEDVQGKNCLTNFHGMDLTRDKMCSMVKKWQTM  120

                                                     MMEIMTPEVQTNDLKDPEVQTNYNSIGK     ACQPIYPLHDVFVR
125  IEAHVDVRTTDGYLLHLFCVGFTKKRNNQIRKTSYAQHQQVRQIRKKMMEIMTPEVQTNDLKDPEVQTNYNSIGKDIEKACQPIYPLHDVFVRKVK  220
     IEAHVDV+TTDGYLL LFCVGFTKKRNNQIRKTSYAQHQQVRQIRKKMMEIMT EVQTNDLK+   +   +SIGKDIEKACQ IYPLHDVFVRKVK
121  IEAHVDVKTTDGYLLRLFCVGFTKKRNNQIRKTSYAQHQQVRQIRKKMMEIMTREVQTNDLKEVVNKLIPDSIGKDIEKACQSIYPLHDVFVRKVK  216

                          ATGDETSAKVERADGYEPPVQESV
              LMELHGEGSSSGR
221  MLKKPKFELGKLMELHGEGSSSGRATGDETSAKVERADGYEPPVQESV  268
     MLKKPKFELGKLMELHGEGSSSG+ATGDET AKVERADGYEPPVQESV
217  MLKKPKFELGKLMELHGEGSSSGKATGDETGAKVERADGYEPPVQESV  264
```

Fig. S15

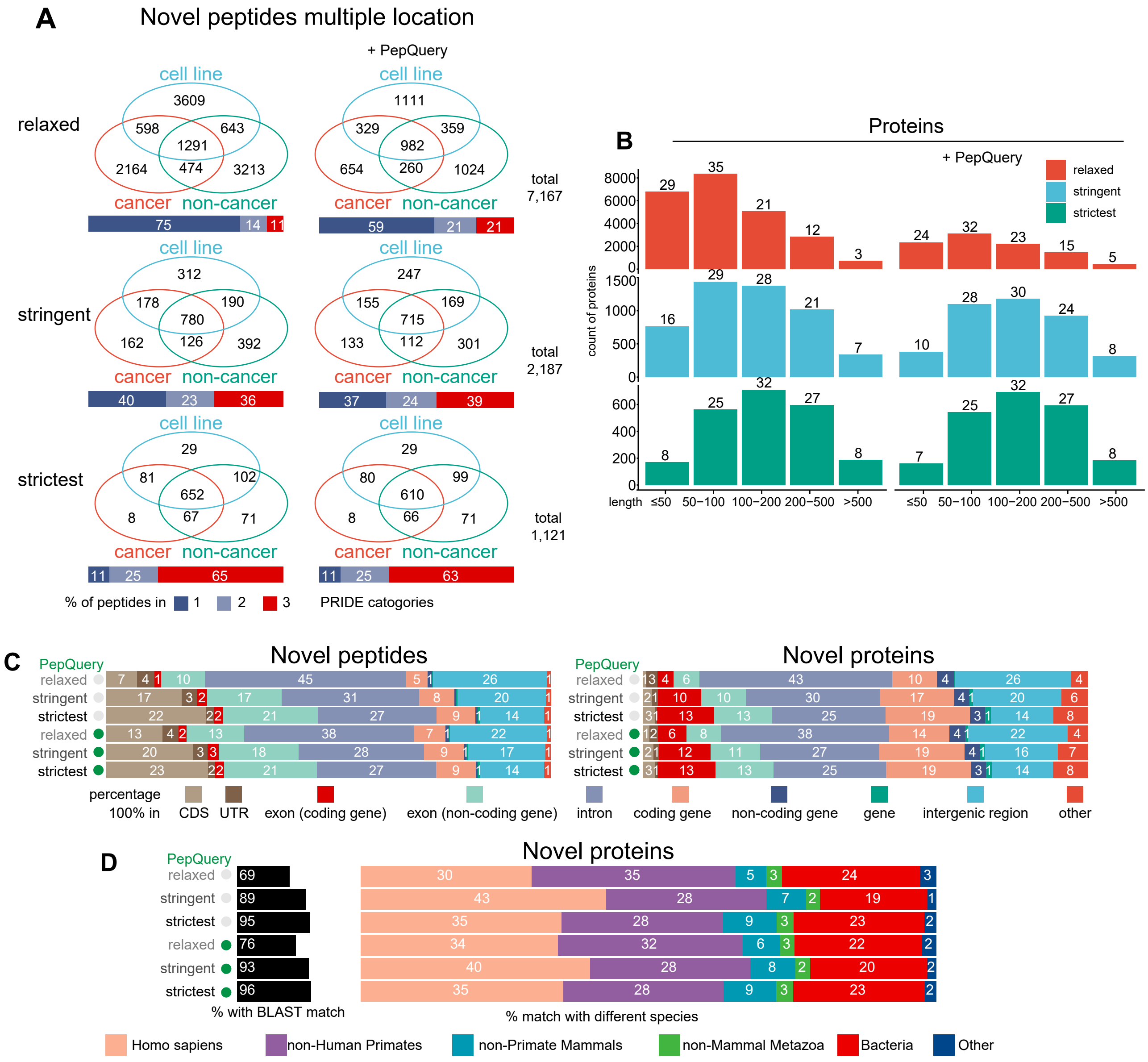
A
Novel peptides multiple location
+ PepQuery
cell line
cancer
non-cancer
relaxed
stringent
strictest
total 7,167
total 2,187
total 1,121
% of peptides in 1 2 3
PRIDE catogories
B
Proteins
+ PepQuery
relaxed
stringent
strictest
count of proteins
length ≤50 50−100 100−200 200−500 >500
C
Novel peptides
Novel proteins
PepQuery
percentage 100% in
CDS
UTR
exon (coding gene)
exon (non-coding gene)
intron
coding gene
non-coding gene
gene
intergenic region
other
D
Novel proteins
% with BLAST match
% match with different species
Homo sapiens
non-Human Primates
non-Primate Mammals
non-Mammal Metazoa
Bacteria
Other

Fig. S16

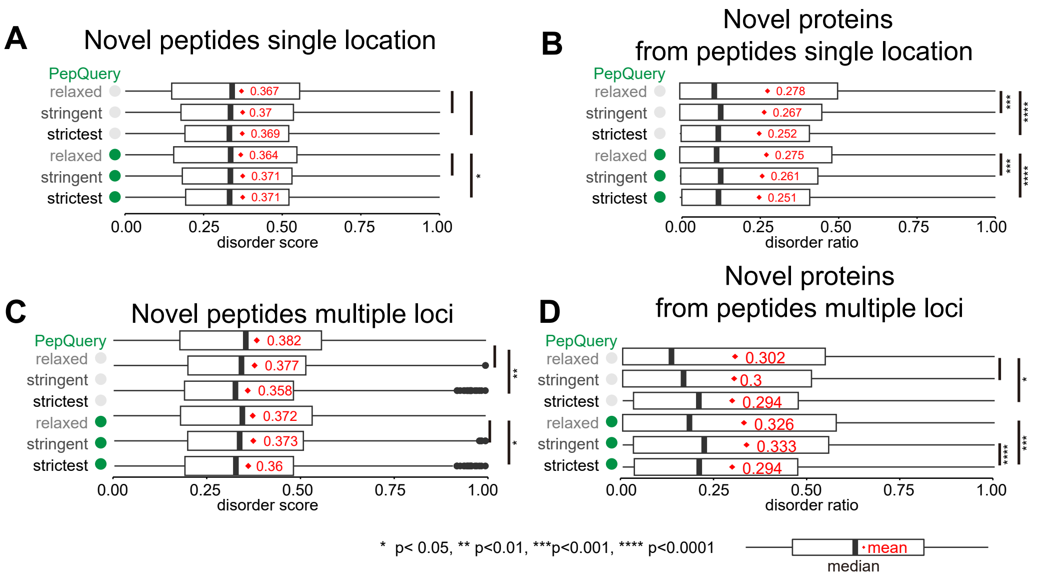

A
Novel peptides single location
PepQuery
relaxed
stringent
strictest
relaxed
stringent
strictest
0.367
0.37
0.369
0.364
0.371
0.371
*
0.00
0.25
0.50
0.75
1.00
disorder score
B
Novel proteins from peptides single location
PepQuery
relaxed
stringent
strictest
relaxed
stringent
strictest
0.278
0.267
0.252
0.275
0.261
0.251
***
****
***
****
0.00
0.25
0.50
0.75
1.00
disorder ratio
C
Novel peptides multiple loci
PepQuery
relaxed
stringent
strictest
relaxed
stringent
strictest
0.382
0.377
0.358
0.372
0.373
0.36
**
*
0.00
0.25
0.50
0.75
1.00
disorder score
D
Novel proteins from peptides multiple loci
PepQuery
relaxed
stringent
strictest
relaxed
stringent
strictest
0.302
0.3
0.294
0.326
0.333
0.294
*
****
***
0.00
0.25
0.50
0.75
1.00
disorder ratio
* p< 0.05, ** p<0.01, ***p<0.001, **** p<0.0001
mean
median
