# Supplementary material for: A Massive Proteogenomic Screen Identifies Thousands of Novel Peptides From the Human “Dark” Proteome
Source: Mol Cell Proteomics. 2024 Jan 17;23(2):100719. doi: 10.1016/j.mcpro.2024.100719 (PMC10867589; doi:10.1016/j.mcpro.2024.100719)
Supplement: Supplementary-information [file mmc10.docx]

# A massive proteogenomic screen identifies thousands of novel peptides from the human “dark” proteome

Supplemental Figure Legend

**Fig. S1** **The percentage of transcripts, exon, and CDS in the human genome based on the GENCODE and GTEx StringTie gene model before and after filtering annotations.** Transcripts annotated on assembled chromosomes were included.

**Fig. S2** **Word clouds of 923 PRIDE project titles (A) and organism parts (B)**. (**A**) The frequencies of words in the titles of the projects were counted and plotted with the Python WordCloud package, excluding common words (i.e., human, proteome, protein, proteins, proteomic, MS, mass, spectrometry, and, of, the, to, for, in, analysis, with, by, proteomics). (**B**) Word cloud of organism parts of the projects. The frequencies of tissues were summed from the counts of spectra of MS files after the first round of MS search. Counts were divided evenly if there were multiple organism parts. NA: organism parts not available.

**Fig. S3** **Numbers of projects (A) and spectra (B) in three major categories of PRIDE projects based on the organism part.** Cancer: body parts from patients with tumors or cultured cells for studying cancer mechanisms. Non-cancer: body parts from individuals without cancer. Cell line: cell lines used to study various biological processes other than cancer mechanisms.

**Fig. S4 Novel peptide counts in different projects.** (**A**) The correlation between novel peptide counts and spectrum counts. Peptides were selected based on the relaxed, stringent, and strictest filtering standards, and with/without PepQuery filtering. Spectrum counts were the number of spectra after quality control in the first round of the MS search. **(B)** Violin plots showing the distribution of percentages of relaxed peptides identified in other filtering standards. Numbers were rounded to the mean percentage values of different PRIDE projects.

**Fig. S5 Length distribution of novel proteins and the longest protein/transcript of each gene**. (**A**) Length distribution of proteins identified from novel peptides with different filtering standards. The y-axis is the count of proteins. The numbers above the bar were the percentage values of that group. One representative protein was selected for each peptide. (**B**) Density plot of transcript length distribution. For each gene, only the longest transcript was used. The x-axis is the length of the transcript and the y-axis is the density. “GTEx all”, all genes from GTEx StringTie gene model; “GTEx filtered”: genes from transcripts detected in ≥ 3 samples and with max TPM ≥ 2 among all samples in GTEx StringTie gene model; “GTEx selected”, genes contributed to protein group N (see “Methods” for detail). “GTEx selected” is different from “GTEx filtered” because “GTEx selected” only includes genes that were predicted coding and contributed to protein group N. “Relaxed”, “Stringent”, and “Strictest”, genes contributed to novel proteins identified under the relaxed, stringent, and strictest standards.

**Fig. S6 Annotation of novel peptides and proteins**. Peptides/proteins were identified based on the relaxed, stringent, and strictest standards. Peptides were not quality controlled by PepQuery. (**A**) Venn diagrams show the number of peptides identified in different PRIDE sample categories. Cancer: body parts from patients with tumors or cultured cells to study cancer mechanisms. Non-cancer: body parts from individuals without cancer. Cell line: cell lines used to study various biological processes other than cancer mechanisms. The percentages of peptides/proteins present in 1, 2, or 3 PRIDE categories were shown in the horizontal bar at the bottom of each Venn diagram. (**B, C**) Relative location of novel peptides (**B**) and proteins (**C**) in different genomic regions. The relative genomic locations of peptides/proteins were determined in the order listed in the figure: CDS, UTR, exon (coding gene), exon (non-coding gene), intron, coding gene, non-coding gene, gene, intergenic region, and other. “exon (coding gene)”: peptide/protein overlaps both “UTR” and “CDS”; “gene”: overlaps both coding and non-coding genes; “other”: overlaps both gene and intergenic region. Only the first genomic location was counted, i.e., peptides grouped as in “CDS” would not be counted as “exon (coding gene)”. (**D**) Percentage of novel proteins with BLAST-matched homologs and the taxonomy distribution of homologs. A single best homologous sequence was selected for each protein and the species were categorized into six groups shown in the figure.

**Fig. S7 PRIDE categories of proteins with different lengths and filtering standards**. Six groups of proteins were identified based on the relaxed, stringent, and strictest standards, with/without PepQuery. For each group, the PRIDE categories of proteins with different lengths were plotted. Numbers inside each bar were percentages.

**Fig. S8 BLAST homologs and the taxonomy distribution of homologs of novel proteins of different lengths under different filtering standards**. Six groups of proteins were identified based on the relaxed, stringent, and strictest standards, with/without PepQuery. For each group, the percentage of BLAST homologs and homologs from different taxonomy of novel proteins with different lengths were plotted. A single best homologous sequence was selected for each protein. The species were categorized into six groups as shown in the figure.

**Fig. S9** **BLAST homologs and the taxonomy distribution of homologs of novel proteins from different PRIDE categories**. Six groups of proteins were identified based on the relaxed, stringent, and strictest standards, with/without PepQuery. For each group, the percentage of BLAST homologs and homologs from different taxonomy of novel proteins with different PRIDE categories were plotted. A single best homologous sequence was selected for each protein. The species were categorized into six groups shown in the figure. CL: cell line; CA: cancer; NC: non-cancer.

**Fig. S10 Length distribution of novel protein with different genomic locations**. Six groups of proteins were identified based on the relaxed, stringent, and strictest standards, with/without PepQuery. The relative genomic locations associated with peptides/proteins were determined in the order listed in the figure. Percentages of proteins with different lengths were labeled inside the bars.

**Fig. S11 PRIDE categories of novel proteins with different genomic locations**. Six groups of proteins were identified based on the relaxed, stringent, and strictest standards, with/without PepQuery. The relative genomic locations of peptides/proteins were determined in the order listed in the figure. Percentages of proteins in different PRIDE categories were labeled inside the bars.

**Fig. S12 BLAST homologs and the taxonomy distribution of homologs of novel proteins with different genomic locations**. Six groups of proteins were identified based on the relaxed, stringent, and strictest standards, with/without PepQuery. Proteins were grouped according to the corresponding genomic locations. The relative genomic locations of peptides/proteins were determined in the order listed in the figure. Percentages of proteins with BLAST-matched homologs and the taxonomy distribution of homologs labeled inside the bars.

**Fig. S13 Density plot of novel proteins identified on different chromosomes**. Proteins were identified based on the relaxed, stringent, and strictest standards with PepQuery filtering. Density plots against each chromosome were drawn with the R package “karyoploteR”. “annotated genes” were from the Bioconductor package “TxDb.Hsapiens.UCSC.hg38.knownGene”.

**Fig. S14 Examples of selected novel proteins**. Novel proteins are located inside the intergenic region (**A**), UTRs (**B**), non-coding exons (**C**), introns (**D**), CDS regions (**E**), and genic and intergenic regions **(F)**. (**D**, **E**, **F**) The upper sequence is from the novel protein, the lower sequence is from homologous proteins. Peptides with MS evidence were in bold red. (**D**, **F**) The middle line is the matched AAs.

**Fig. S15 Annotation of peptides with multiple genomic loci**. (**A**) Novel peptides identified in different PRIDE sample categories. Peptides were analyzed by the three filtering standards (relaxed, stringent, and strictest), with or without the PepQuery quality control (see Methods for detail). Total counts of peptides were labeled. The percentage of peptides present in 1, 2, or 3 PRIDE categories are shown at the bottom of each plot. (**B**) Length distribution of proteins with/without PepQuery. The numbers above the bar were percentage values of that group. The x-axis is the length of proteins and the y-axis is the count of proteins. (**C**) Relative abundance of novel peptides and proteins selected in different standards in different genomic regions. **(D)** Percentage of novel proteins with BLAST-matched homologs and the taxonomy distribution of homologs. For each locus of peptides, a single representative protein was selected.

**Fig. S16 Disordered features of peptides and proteins**. (**A**, **C**) Boxplots of average disorder score calculated by IUPred3 from novel peptides with single genomic locus (**A**) and multiple loci (**C**). (**B**, **D**) Boxplots depicting the ratio of disordered residues predicted of each protein relative to the protein’s length based on peptides with single genomic locus (**B**) and multiple loci (**D**). Residues with a score > 0.5 were considered disordered. In the box-whisker plot, the dark vertical lines indicated the median values; Red numbers were mean values of the ratios of disordered residues in protein sequences.

Supplemental Tables

Table S1

Table S1 Known human gene model download links

Table S2

Table S2 Summary information for the 923 PRIDE projects

Table S3

Table S3 List of the MS runs used in this work.

Table S4

Table S4 Identified novel peptides and their annotations. Only peptides with single genomic location were included.

Table S5

Table S5 Identified novel proteins and their annotations. Only proteins from peptides with single genomic location were included.

Table S6

Table S6 Summary of transcripts from GENCODE and circRNA identified with novel peptides or proteins.

Table S7

Table S7 Summary of novel protein domain structures.

Table S8

Table S8 Selected peptides with multiple genomic locations.
